# Spatio-temporal regulation of nuclear division by Aurora B kinase Ipl1 in *Cryptococcus neoformans*

**DOI:** 10.1371/journal.pgen.1007959

**Published:** 2019-02-14

**Authors:** Neha Varshney, Subhendu Som, Saptarshi Chatterjee, Shreyas Sridhar, Dibyendu Bhattacharyya, Raja Paul, Kaustuv Sanyal

**Affiliations:** 1 Molecular Mycology Laboratory, Jawaharlal Nehru Centre for Advanced Scientific Research, Jakkur, Bangalore, India; 2 Department of Solid State Physics, Indian Association for the Cultivation of Science, Kolkata, India; 3 Tata Memorial Centre, Advanced Centre for Treatment Research and Education in Cancer, Kharghar, Navi Mumbai, India; Duke University, UNITED STATES

## Abstract

The nuclear division takes place in the daughter cell in the basidiomycetous budding yeast *Cryptococcus neoformans*. Unclustered kinetochores gradually cluster and the nucleus moves to the daughter bud as cells enter mitosis. Here, we show that the evolutionarily conserved Aurora B kinase Ipl1 localizes to the nucleus upon the breakdown of the nuclear envelope during mitosis in *C*. *neoformans*. Ipl1 is shown to be required for timely breakdown of the nuclear envelope as well. Ipl1 is essential for viability and regulates structural integrity of microtubules. The compromised stability of cytoplasmic microtubules upon Ipl1 depletion results in a significant delay in kinetochore clustering and nuclear migration. By generating an *in silico* model of mitosis, we previously proposed that cytoplasmic microtubules and cortical dyneins promote atypical nuclear division in *C*. *neoformans*. Improving the previous *in silico* model by introducing additional parameters, here we predict that an effective cortical bias generated by cytosolic Bim1 and dynein regulates dynamics of kinetochore clustering and nuclear migration. Indeed, *in vivo* alterations of Bim1 or dynein cellular levels delay nuclear migration. Results from *in silico* model and localization dynamics by live cell imaging suggests that Ipl1 spatio-temporally influences Bim1 or/and dynein activity along with microtubule stability to ensure timely onset of nuclear division. Together, we propose that the timely breakdown of the nuclear envelope by Ipl1 allows its own nuclear entry that helps in spatio-temporal regulation of nuclear division during semi-open mitosis in *C*. *neoformans*.

## Author summary

Unlike the model ascomycetous budding yeast *Saccharomyces cerevisiae*, microtubule organizing centers (MTOCs) coalesce to form the spindle pole body (SPB) in *C*. *neoformans*. This process also ensures unclustered kinetochores to gradually cluster in this organism. As *C*. *neoformans* cells enter mitosis, the nuclear envelope ruptures and the nucleus eventually moves to the daughter bud before division. Here, we combine cell and systems biology techniques to understand the key determinants of nuclear division in *C*. *neoformans*. We show that the evolutionarily conserved Aurora B kinase Ipl1 enters the nucleus during the mitotic phase as cells undergo semi-open mitosis. Ipl1 regulates dynamics of cytoplasmic microtubules, cytosolic proteins such as Bim1 and dynein-mediated cortical forces and integrity of the nuclear envelope to ensure timely kinetochore clustering and nuclear division in this medically relevant human pathogenic budding yeast.

## Introduction

High-fidelity chromosome segregation ensures faithful transmission of the genetic material to subsequent generations. This process is powered by the dynamic interactions of the centromere-kinetochore complex and the mitotic spindle. The microtubule organizing centers (MTOCs), microtubule fibers, microtubule-associated proteins (MAPs) and molecular motors belonging to kinesin and dynein superfamilies influence microtubule (MT) dynamics and functioning of the mitotic spindle. Among the three types of MTs emanating from MTOCs, the cytoplasmic MTs (cMTs) ensure proper spindle positioning and nuclear migration [1, 2]. Several MAPs and MT-based motor proteins participate in organizing the MT cytoskeleton by regulating polymerization-depolymerization kinetics, dynamics of cross-linking, and motility of MT fibers [3, 4]. The plus-end tracking proteins (+TIPs), a subgroup of MAPs is characterized by their preferential association with the MT plus-ends. Among the plethora of +TIPs, Bim1, the EB1 homolog in yeast, localizes at the distal ends of cMTs, establishes contacts with the cortical polarity determinant Kar9 and guides the plus-ends of cMTs along the cortical actin cables for penetration of MTs into the budding daughter cell [5, 6]. In contrast to Kar9-mediated interactions, dynein molecules are transported from the less dynamic minus-end towards the plus-end of MTs, empowering spindle movement through the mother-daughter bud neck [7, 8]. Perhaps, dynein molecules undergo spatio-temporal regulation during spindle movement by regulated targeting of She1 to cMTs [9]. In addition, cytoplasmic dynein molecules in yeast can also potentially crosslink two MT fibers physically and slide them along each other [10].

An interplay between kinases and phosphatases spatio-temporally regulate kinetochore-MT interactions (reviewed in [11]). The evolutionarily conserved Ipl1/Aurora B kinase, the best-studied regulator of the kinetochore-MT attachments, primarily senses the tension at the kinetochore [12, 13]. Aurora B kinase Ipl1 stabilizes bi-oriented kinetochore-MT attachments and destabilizes incorrect kinetochore-MT attachments [14, 15]. In addition, Ipl1 ensures the integrity and timely disassembly of the mitotic spindle by phosphorylating She1 and Bim1 respectively in *Saccharomyces cerevisiae* [16–18]. Ipl1 regulates dynein activity along the cMTs by phosphorylating She1 and influences movement of the pre-anaphase spindle into the mother-daughter bud neck [8].

Unlike hemiascomycetous budding yeasts such as *S*. *cerevisiae*, the process of chromosome segregation is less known in the phylum Basidiomycota [19, 20] that shared a common ancestor with Ascomycota more than 500 million years ago [21]. The best-studied basidiomycete is the human pathogen *Cryptococcus neoformans*, that causes life-threatening pneumonia and meningitis, and is the 5^th^ most leading cause of death in immunocompromised patients [22]. Azole-associated acquisition of aneuploidy is a well-elucidated mechanism that contributes to the emergence of transient azole resistance in *C*. *neoformans* [23, 24]. Clones that emerged at the highest drug concentration tested were found to be disomic for multiple chromosomes [24]. Thus, aneuploidy provides an increased fitness to *C*. *neoformans* under the azole stress [25].

Although *C*. *neoformans* divides by budding, a number of striking variations are observed in the dynamics of MTOCs, the site of nuclear division and the timing of kinetochore clustering as compared to the ascomycetes such as *S*. *cerevisiae* and *C*. *albicans*. In ascomycetous budding yeast species, a single MTOC, also known as the spindle pole body (SPB), is embedded in the nuclear envelope (NE) [26]. Once the SPB is duplicated during the G1/S transition phase, the kinetochore-MT interaction is established although the NE never breaks down, resulting in a closed mitosis [27, 28]. In contrast, *C*. *neoformans* cells have several MTOCs present throughout the cytoplasm during interphase and undergo semi-open mitosis characterized by transient rupture of the NE during metaphase to anaphase transition [20, 29]. In ascomycetes, the nucleus migrates close to the mother-daughter cell junction and divides into two equal halves [19, 30], while in *C*. *neoformans*, the nucleus first migrates completely to the daughter cell and divides [19, 20]. The kinetochores are clustered and tethered to SPBs by MTs during most of the cell cycle in ascomycetous yeast species [28, 31]. In contrast, the unclustered kinetochores gradually coalesce into a single cluster with the help of MTs as cells progress towards mitosis in *C*. *neoformans* [20]. We previously demonstrated that these fundamental variations in the process of nuclear division in these two fungal phyla are determined by the populations of cMTs and cortical dyneins [19]. Here, we combined cell biology studies and computational simulations to understand the molecular basis of unconventional nuclear division in *C*. *neoformans*. We used the ploidy sensor Ipl1 as a tool to study this process. Overall, our results uncover a distinct mechanism leading to atypical mitosis and the role of Ipl1 in this process to maintain ploidy in a basidiomycete *C*. *neoformans*.

## Results

### Timely and regulated nuclear entry of cytosolic Ipl1 during semi-open mitosis in *C*. *neoformans*

**Fig 1:**
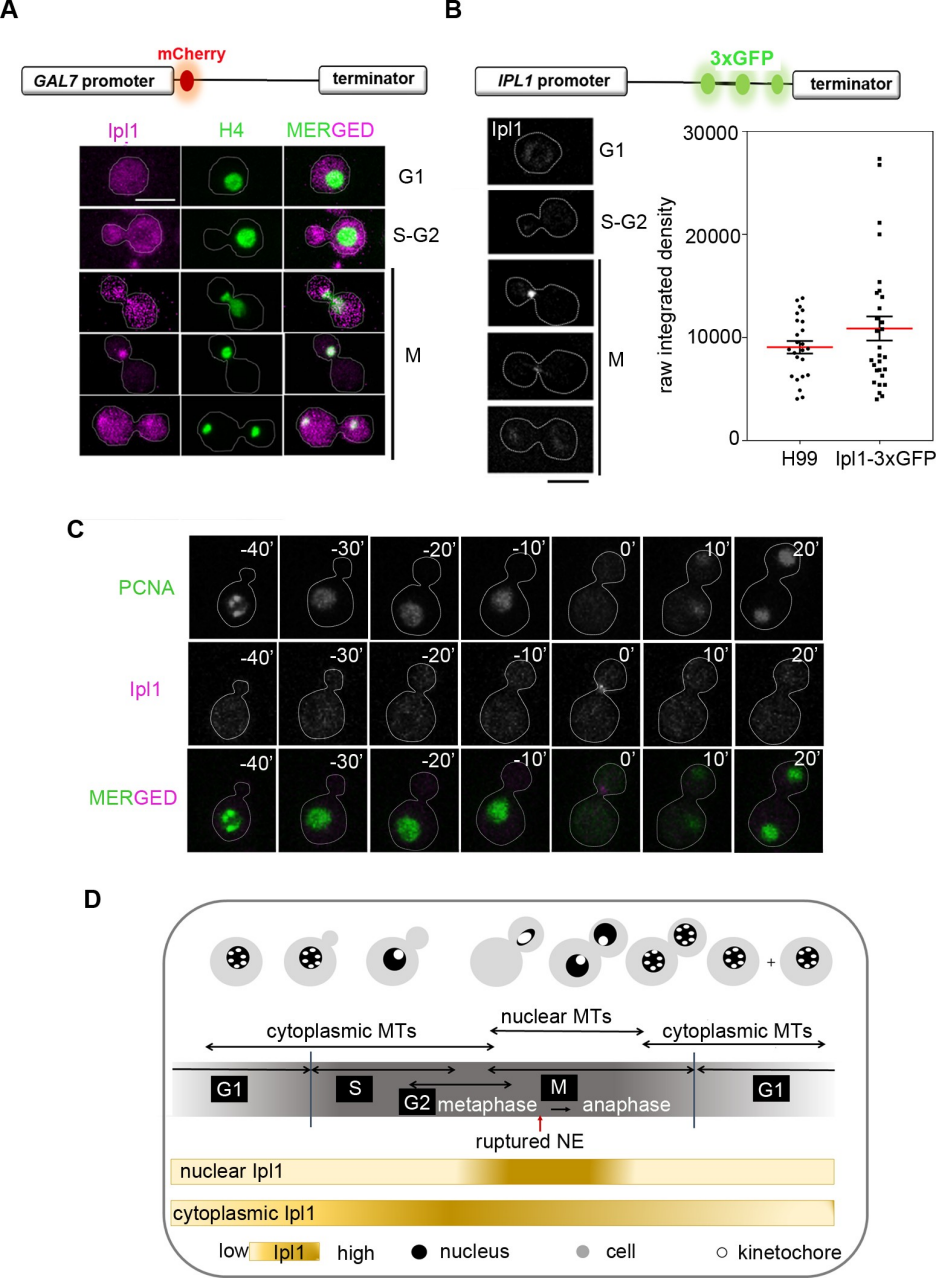
Nuclear entry of Ipl1 during semi-open mitosis in *C*. *neoformans*. (**A)** CNNV114 cells co-expressing mCherry-Ipl1 and histone H4-GFP depicting localization of Ipl1 and histone H4 respectively during mitosis. Bar, 5μm. (**B)** Localization of Ipl1 in the wild-type CNNV113 cells expressing Ipl1-3xGFP in interphase and mitotic cells. Bar, 5μm (left). Quantification of the total raw integrated density values of GFP signal from the pre-mitotic cells from the strain H99 and CNNV113 (n>25). Mean (red line) and SEM values are shown. (**C**) Time-lapse snapshots of wild-type CNNV112 cells at the indicated time interval in the permissive conditions. The nucleus is marked by PCNA-GFP which leaks into the cytosol upon the breakdown of the nuclear envelope. Ipl1 is marked by mCherry which is pseudo-colored as magenta. Bar, 5μm. (**D)** A graphical summary showing cell cycle-dependent regulation of Ipl1 in the wild-type cells.

The ORF CNAG_01285 is the homolog of Ipl1 in *C*. *neoformans* in the FungiDB (http://fungidb.org/fungidb/) having an evolutionarily conserved kinase domain (**S1A and S1B Fig**). To study the localization of Ipl1, we functionally expressed it as a fusion protein with mCherry at its N-terminus under the *GAL7* promoter in the strain CNNV114 co-expressing GFP-tagged histone H4. Strikingly, overexpressed Ipl1 shows a distinct localization to the cytosol throughout the cell cycle. However, Ipl1 is also nuclear localized only during mitosis (**Fig 1A**). Ipl1 colocalizes with GFP-tagged histone H4 from the time of migration of nucleus to the daughter bud till the nucleus is divided into two equal halves. We further validated the localization of Ipl1 when expressed at the cellular level by functionally expressing it as a fusion protein with a triple GFP epitope at its C-terminus under the native promoter in the strain CNNV113. A reduced Ipl1 localizes to the nucleus only during specific stages of mitosis. The cytosolic signal is barely visible possibly due to low and dispersed signal intensities spread across the cytoplasm (**Fig 1B**). In fact, Ipl1’s localization in the nucleus at certain stages of the cell cycle also remained undetected when expressed under the native promoter. While nuclear localization of Ipl1 in mitosis is evolutionary conserved, its cytosolic localization remained speculative in *S*. *cerevisiae* [32] and unknown in other yeast species.

The NE transiently ruptures during mitosis in *C*. *neoformans* [20]. To test whether rupturing of the NE during mitosis helps in nuclear entry of Ipl1, we tagged nuclear localized proliferating cell nuclear antigen (PCNA), an S-phase specific nuclear protein, with GFP in CNNV112 cells expressing mCherry-Ipl1. Strikingly, Ipl1 shows a distinct cytosolic localization throughout the cell cycle (**S2A Fig**). However, during disassembly of the NE in mitosis marked by the diffusion of nuclear PCNA to the cytosol, Ipl1 is also found to be nuclear localized, suggesting rupturing of the NE during mitosis helps in the timely nuclear entry of Ipl1. Further, cytosolic and nuclear localization dynamics of Ipl1 was examined by performing time-lapse microscopy with CNNV112 cells grown in permissive conditions (**Fig 1C, S1 movie**). At the time of nuclear migration to the daughter bud, Ipl1 gains access to the nucleus due to the NE rupture and thus localized both in the cytosol and as well as in the nucleus. This suggests that the NE rupture event facilitates the nuclear Ipl1’s tension sensing function during kinetochore-MT attachments. In addition, cytosolic localization of Ipl1 throughout the cell cycle is possibly important for migration of the nucleus from the mother to the daughter bud. Incidentally, depletion of Ipl1 resulted in a premature rupture of the NE and leakage of PCNA into the cytosol in 18% of budded population indicating that Ipl1 also regulates the timely disassembly of the NE to facilitate its own entry into the nucleus during mitosis (**Figs S2B and 1D**).

### Ipl1 is essential for viability and it regulates the dynamics of nuclear division

**Fig 2:**
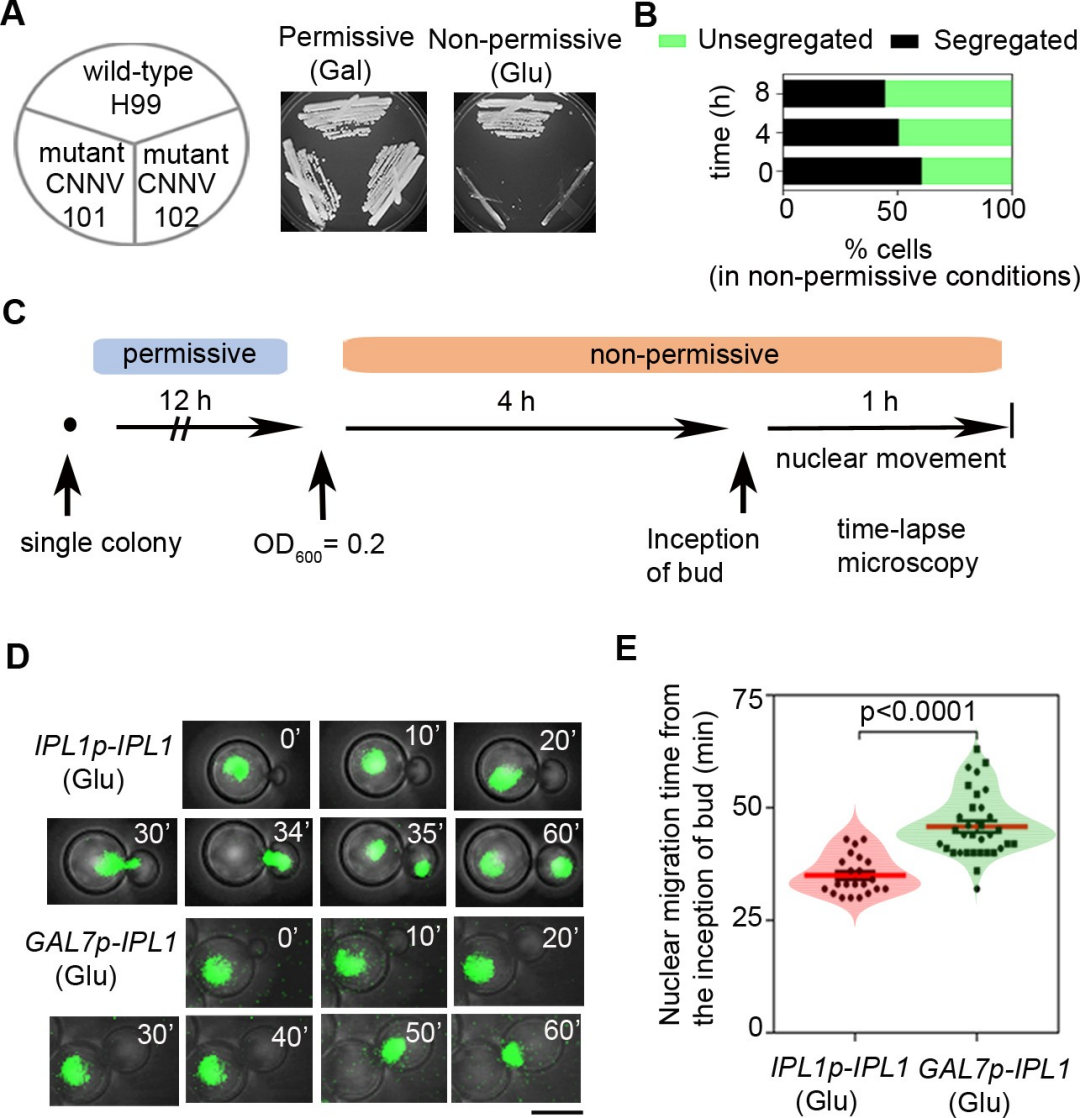
Ipl1 is required for the cell viability and timely nuclear division in *C*. *neoformans*. **(A)** *C*. *neoformans* cells expressing Ipl1 either from its native promoter (H99) or the *GAL7* promoter (CNNV101 and CNNV102) were streaked on plates containing indicated media for 2 to 3 days at 30°C. (**B)** The Ipl1-depleted cells of strain CNNV104 having the budding index (B.I.) ≥ 0.4 grown for an indicated time in non-permissive conditions were analyzed for nuclear segregation. (**C)** Experimental design showing steps followed for capturing time-lapse movies of nuclear migration. (**D)** Time-lapse snapshots of wild-type CNVY108 and Ipl1-depleted strain CNNV104 at the indicated time in each case. The nucleus is marked by histone H4-GFP. Bar, 5μm. (**E**) Quantification of the nuclear migration time till anaphase completion in the wild-type (n = 22) and Ipl1-depleted (n = 32) cells. Mean (red line) and SEM values are shown; p<0.0001; unpaired *t*-test.

To study the essentiality of *IPL1* for cell viability, we constructed the conditional promoter shut-down mutant strain by placing the ORF under the regulatable *GAL7* promoter which is repressed in the presence of glucose (non-permissive) but expressed when galactose (permissive) is used as the carbon source [33]. The inability of two independent conditional *ipl1* mutants, CNNV101 and CNNV102, to grow under non-permissive conditions confirmed that Ipl1 is essential for viability in *C*. *neoformans* (**Fig 2A**). Mutants of *ipl1* were first identified in the *i*ncrease-in-*pl*oidy screen of mutants in *S*. *cerevisiae*, an ascomycete [34]. To test this function of Ipl1 in *C*. *neoformans*, a basidiomycete yeast, we performed imaging of CNNV114 cells co-expressing Ipl1-mCherry and histone H4-GFP. Although Ipl1-mCherry signals could not be detected after growth of cells in non-permissive conditions for 4 h (**S3A Fig**), Ipl1-depleted cells exhibited the most severe phenotype of accumulation of cells having unsegregated nuclei (56% of the population) after 8 h of growth under non-permissive conditions (**Fig 2B**). Therefore, we performed subsequent experiments by growing the conditional *ipl1* mutant in non-permissive conditions for 8 h. To test the occurrence of unsegregated nuclei in the Ipl1-depleted cells, we imaged nuclear migration in the live wild-type CNVY108 and mutant CNNV104 cells (**Fig 2C**) that suggested while a fraction of Ipl1-depleted cells had the nucleus remained unsegregated in the mother cell or present at the mother-daughter cell junction, a proportion of the mutant cells had nuclei segregated with a delay in the nuclear migration (mean = 47 min) as compared to the wild-type cells (mean = 35 min) (**Fig 2D and 2E; S2 Movie**). Taken together, we conclude that Ipl1 is required for timely nuclear migration during cell division in *C*. *neoformans*.

### Ipl1 maintains spatio-temporal regulation of dynamic kinetochore-MT interactions

**Fig 3:**
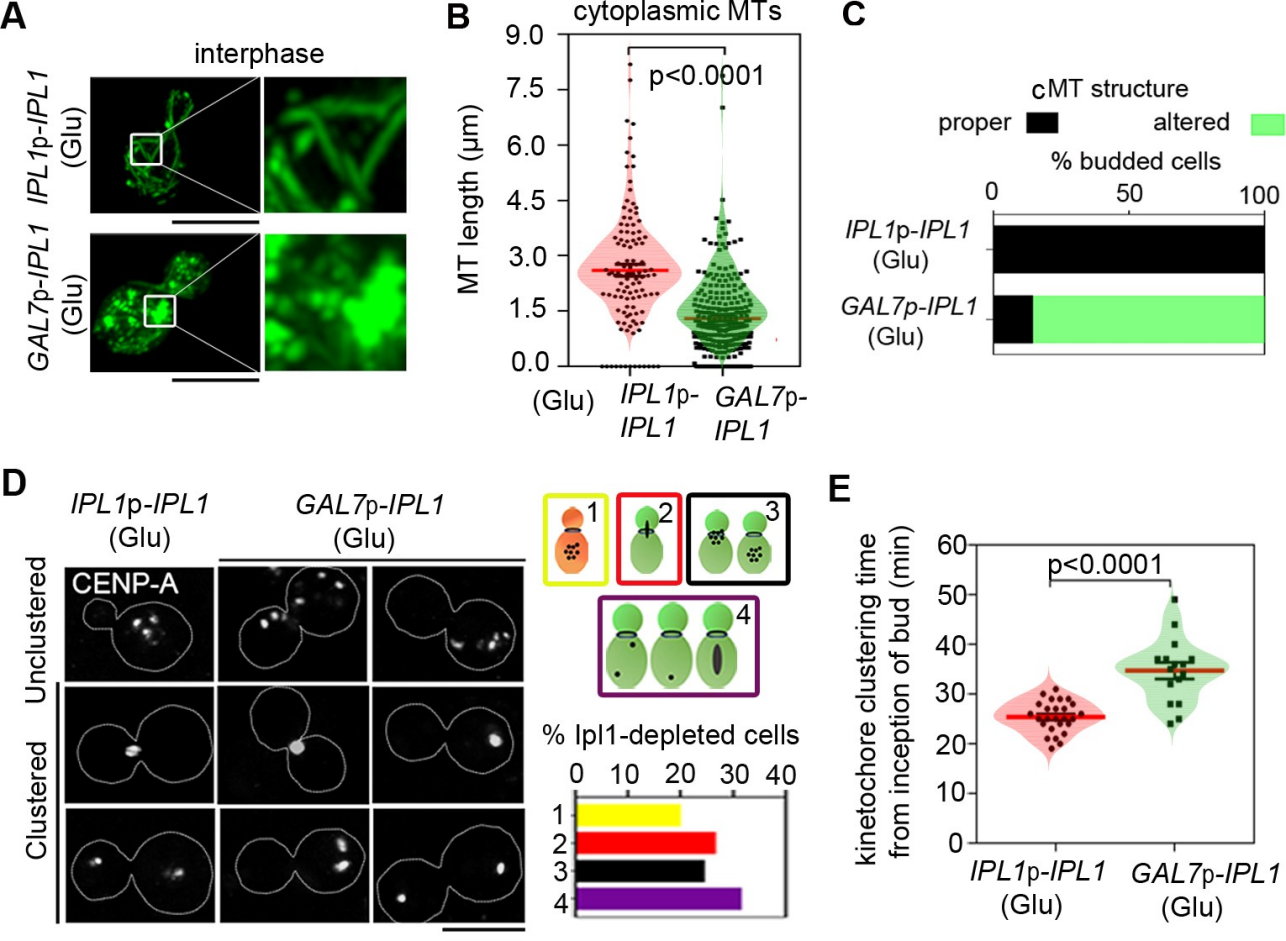
Ipl1 spatio-temporally regulates kinetochore-microtubule interactions. **(A)** Morphology of MTs in the wild-type CNVY107 and Ipl1-depleted CNNV105 cells at interphase expressing GFP-Tub1. Corresponding zoomed images are also shown. Bar, 5μm. (**B)** Quantification of the cMT length in the budded wild-type (n = 171) and Ipl1-depleted cells (n = 318). Mean and SEM values are marked; p<0.0001, unpaired *t*-test. (**C)** Percentages of cells with intact and disintegrated MTs in the wild-type and mutant cells (n = 96). (**D**) Observed phenotypes of kinetochore clustering in the wild-type and Ipl1-depleted cells (left). Quantification of the wild-type-like (1) and defective phenotypes of unclustered/clustered kinetochores (2–4) observed in the Ipl1-depleted cells is represented as a bar graph (n = 94). Bar, 5μm (right). (**E)** Quantification of the kinetochore clustering time (min) in wild-type (n = 26) and Ipl1-depleted cells (n = 18).; p<0.0001, unpaired *t*-test.

Nuclear migration from the mother cell to the daughter is governed by cMTs in yeasts [2, 19, 35]. Therefore, we analyzed the length of the cMTs in Ipl1-depleted cells. The length of the cMTs was severely affected in the mutant CNNV105 cells with an average length of the cMTs of 1.24 μm as compared to 2.7 μm found in the wild-type CNVY107 cells (**Fig 3A and 3B**). More than 80% budded Ipl1-depleted cells possessed shorter MTs as opposed to the majority of the cells having intact MTs in the wild-type (**Fig 3C**), although the total cellular pool of tubulin remains unaltered even after depletion of Ipl1 (**S3B Fig**).

Co-localization of MTs with kinetochores as well as aberrant kinetochore clustering in response to nocodazole suggested that MTs are indispensable for kinetochore clustering in *C*. *neoformans* [20]. We investigated kinetochore clustering in Ipl1-depleted cells having compromised integrity of MTs by localizing mCherry-CENP-A signals (inner kinetochore) in the wild-type, CNVY107 and Ipl1-depleted strain CNNV105 (**Fig 3D**). Approximately equal proportion of Ipl1-depleted cells exhibited four distinct phenotypes (a) unclustered kinetochores in the mother cell with no signal in the daughter cell (ratio of the daughter/mother cell size < 0.4) (19%), (b) unclustered kinetochores in the mother cell with no signal in the daughter cell (ratio of the daughter/mother cell size > 0.5) or unclustered kinetochore at the neck of mother-daughter cell (24%), (c) clustered kinetochores that were stuck at the neck of the mother-daughter cell junction (26%), and (d) unsegregated or aberrantly segregated kinetochores (31%) between the mother and daughter cells (**Fig 3D**). While the kinetochores were found to be unclustered in the wild-type cells with an average ratio of the daughter /mother cell size of 0.4, kinetochores remained unclustered in Ipl1-depleted cells at a much higher average ratio of 0.75 (**S3C Fig**), indicating a delay in kinetochore clustering in the Ipl1-depleted cells. Thus, we quantified the time required for kinetochore clustering in the wild-type, CNVY107 and the Ipl1-depleted mutant strain CNNV105 (**Fig 3E**). Strikingly, the Ipl1-depleted cells displayed a 10 min delay in time (35 min) required for kinetochore clustering as compared to the wild-type (25 min). It is noteworthy that not all the cells with disintegrated MTs harbor defective kinetochore clusters in the mutant (**S3D Fig**). Since an outer kinetochore protein Dam1 was shown to have a compromised localization in the absence of the mitotic spindle in *S*. *cerevisiae* [36, 37], we examined the localization of two outer kinetochore proteins, Mtw1 and Dad1, in CNNV108 and CNNV109 respectively. Localization of both the proteins was apparently unaltered in the Ipl1-depleted cells (**S3E Fig**). Next, we compared the delayed kinetochore clustering phenotype observed in the Ipl1-depleted cells with the wild-type cells treated with the MT-depolymerizing drug such as nocodazole. While the kinetochores were found to be unclustered in the wild-type cells with an average ratio of the daughter /mother cell size of 0.5, kinetochores remained unclustered in nocodazole-treated cells at a higher average daughter /mother cell size of 0.86 (**S4A and S4B Fig**), similar to the Ipl1-depleted cells. Together, these results confirmed that without affecting the kinetochore localization, depletion of Ipl1 affects the MT stability resulting in an alteration in spatio-temporal regulation of kinetochore clustering in *C*. *neoformans*. Thus, we focussed our subsequent studies on how Ipl1 might be regulating kinetochore clustering and nuclear migration in *C*. *neoformans*.

### The cortical interaction of cMTs with an initial bias is required for MTOC clustering and nuclear migration

**Fig 4:**
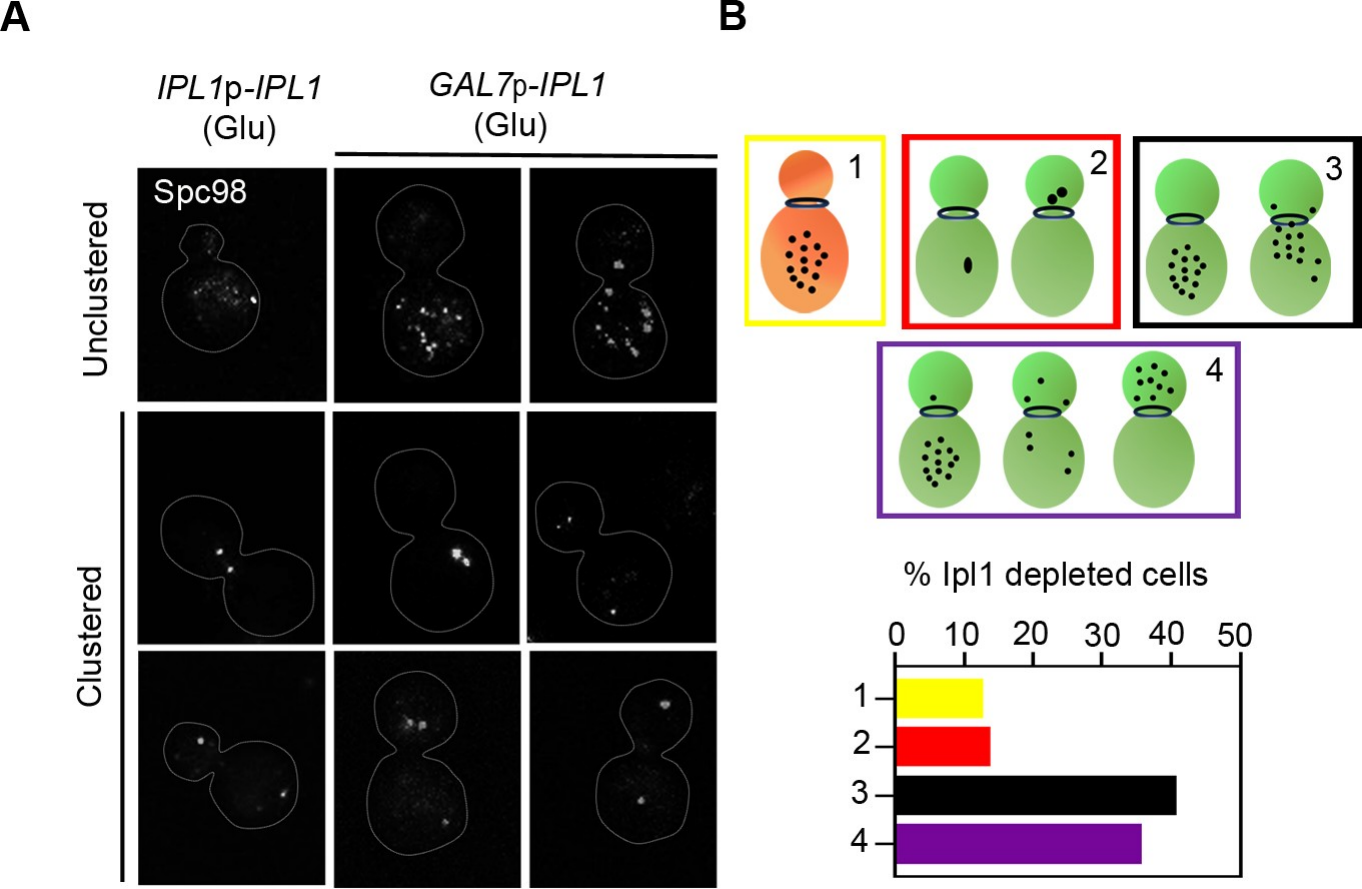
Dynamics of MTOC clustering in the wild-type and Ipl1-depleted cells. **(A)** Images of different phenotypes of MTOC (Spc98-3xGFP) clustering in the CNNV118 cells grown in permissive and non-permissive conditions. **(B)** Quantification of the wild-type-like (1) and defective phenotypes of unclustered/ clustered MTOCs (2–4) observed in the Ipl1-depleted cells is represented as a bar graph (n = 70). Bar,10 μm.

**Fig 5:**
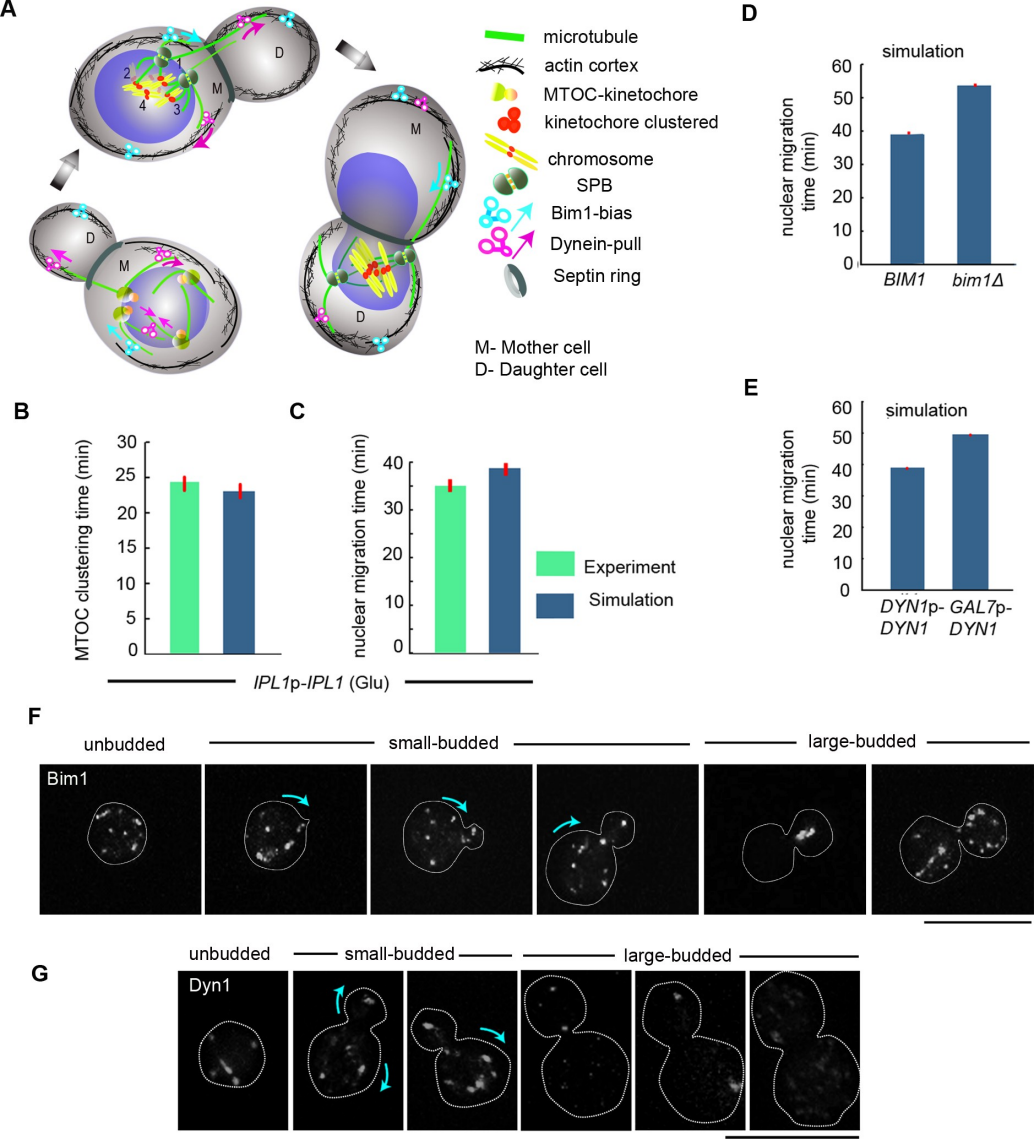
Bim1-dynein-mediated effective bias ensures timely MTOC clustering and nuclear migration. **(A)** Schematic of MTOC clustering, nuclear migration, biorientation of chromosomes and possible chromosomal attachments. Arrows within a cell indicate direction forces required for nuclear migration and MTOC clustering. Interactions of cMT plus-ends with cortical dynein (CI), Bim1-driven bias of the cMT plus-end (CIB) and MT sliding by crosslinking dyneins on the nuclear surface (NI) can independently lead to MTOC clustering. (**B-C)** Mean MTOC clustering and nuclear migration time since bud emergence in presence of CIB (n≥31). (**D)** The nuclear migration time is prolonged in the *bim1*Δ cells as compared to the wild-type cells. (**E)** An enhanced dynein activity delays nuclear migration. **(F)** Localization of Bim1 in the CNNV119 cells expressing Bim1-3xGFP in interphase and mitotic cells. Bar, 10μm. Arrow within a cell indicates the direction of Bim1-mediated force. **(G)** Localization of Dyn1 in the CNNV116 cells expressing Dyn1-3xGFP in interphase and mitotic cells. Bar, 10μm. Arrow within a cell indicates the direction of Dyn1-mediated force.

MTOCs conglomerate into a compact mass to form the fully functional mother SPB [38, 39], which simultaneously facilitates the clustering of MTOC-bound kinetochores in *C*. *neoformans* [40]. To elucidate the Ipl1-mediated process of kinetochore clustering, we studied the dynamics of MTOCs by localizing Spc98, an MTOC component, in the strain CNNV118 (**Fig 4**). In unbudded interphase cells, MTOCs are unclustered and puncta of Spc98-3xGFP signals are scattered (**Fig 4A, left panels**). As the cell cycle progresses, Spc98-3xGFP signals gradually cluster to form a mature SPB. Subsequently, signals representing clustered MTOCs segregate into two halves during mitosis, one of which is retained in the daughter bud and the other is delivered to the mother cell (**Fig 4A, left panels**). Next, we investigated MTOC clustering in Ipl1-depleted CNNV118 cells by localizing Spc98-3xGFP upon their growth in non-permissive conditions for 8 h (**Fig 4A, right panels)**. The Ipl1-depleted cells exhibited four distinct phenotypes (a) unclustered MTOCs in the mother cell with no signal in the daughter cell (ratio of the daughter/mother cell size < 0.4) (12%), (b) unclustered MTOCs in the mother cell with no signal in the daughter cell (ratio of the daughter/mother cell size > 0.5) or unclustered MTOCs scattered at the neck of mother-daughter cell (40%), (c) clustered MTOCs or clustered segregated SPBs present either in the mother or daughter cell (12%), and (d) unsegregated or aberrantly segregated SPBs (34%) between the mother and daughter cells (**Fig 4B**). While the MTOCs were found to be unclustered in the wild-type cells with an average ratio of the daughter /mother cell size of 0.4–0.5, MTOCs remained unclustered in Ipl1-depleted cells at an average ratio of >0.5, indicating a delay in MTOC clustering in the Ipl1-depleted cells. Further, using an *in silico* model, we canvassed various plausible MT-driven MTOC clustering schemes (**Fig 5A**) characterized by parameters (**Table 1**) [41, 42]. We assumed that a single kinetochore is associated with a single MTOC prior to the clustering. The cortical interaction of the cMTs mediated by dyneins and other motor proteins generates a directed mechanical force on various intracellular components including MTOCs [43–45]. As an additional component to cortical interaction, we introduced a form of bias on the cortically interacting cMT plus ends towards the septin ring that is triggered by Bim1 yielding an enhancement in the force transmission to the MTOCs and leading to a complete fusion (~98%) of MTOCs within 23 min (**Fig 5B**). Migration of the nucleus from the mother to the daughter cell is partially concomitant with the process of MTOC clustering during mitosis in *C*. *neoformans* [20]. Henceforth, it is essential to validate whether the proposed clustering scheme adequately corroborates with the timely nuclear migration [6, 43]. We found that the recruitment of cortical interaction with bias at the onset of the MTOC clustering and sustaining it all through takes ~38 min for proper nuclear migration (**Fig 5C**), close to the experimental time scale of ~35 min. Hence, we employed the cortical interaction with bias scheme to explore the attributes of the mitotic cell cycle in the wild-type (**S3A Movie**).

**Table 1:** Various parameters used to develop the computational model.

Abbreviation	Meaning	Value	Reference
*N*_KT_	Number of KTs	14	[19]
*r*_mother_	Radius of the mother cell	3.0 μm	This study
*r*_nuc_	Radius of the Nucleus	1.0 μm	This study
*r*_SPB_	Radius of single SPB	0.125 μm	[46, 47]
*K*_cor_	Spring constant of the cortex	5.0 pN/μm	[19, 48]
*η*_cyt_	Viscosity of cytoplasm	5.0 pN s/μm^2^	[48]
*η*_nu_	Viscosity of nucleoplasm	10.0 pN s/μm^2^	[48]
*η*_NE_	Effective viscosity of NE	10.0 pN s/μm^2^	[48]
*v*_g_, *v*_s_	MT’s growth, shrink velocity	10.4 μm min^-1^, 28.6 μm min^-1^	[49, 50]
*f*_c_	Catastrophe frequency of MT	1.0 min^-1^ (wild-type), 1.0–21.0 min^-1^ (mutant)	[49, 50]
*f*_r_	Rescue frequency of MT	0.02 min^-1^	[49, 50]
*f*_c_^stall^	Catastrophe rate of stalled MT	0.04 s^-1^	[51]
*f*_stall_	MT's stall force	1.7 pN	[52]
*f*_dyn_	Force produced by single dynein	1.0 pN	[53, 54]
*λ*_dyn_	Number of dynein per unit length per MT	6.0 μm^-1^	[55]
*λ*_ipMT_	Number of ipMT motor per unit length per MT	1.0 μm^-1^	[48]
*f*_kinesin-5_	Force produced by single kinesin-5 motor	1.0 pN	[19]
*K*_cohesion_	Spring constant of the cohesion springs	0.1 pN/μm	[56]
*K*_C_	Spring constant of the KT–kMT attached springs	10.0 pN/μm	[48, 57]
*K*_fibril_	Spring constant of the KT fibril	5.0 pN/μm	[48, 57]

### Cortical recruitment of Bim1 as a bias generating component with dynein provides an effective cortical bias essential for kinetochore clustering and nuclear migration

The cMTs are required to be long enough to establish contacts with the cell cortex to render the directional preference of cMTs via cortical interaction with bias [58, 59]. A combined action of Bim1 and dynein has been shown to be essential to impart an ‘effective’ cortical bias on the cMT plus-ends penetrating into the cortex towards the septin ring [19, 60]. It has been reported previously that *C*. *neoformans* lacks a Kar9 homolog (**Table 2**) [61], we probed for the function of Bim1 and dynein in generating an effective cortical bias at the plus-ends of the cMTs in *C*. *neoformans*. We assumed a uniform distribution of dynein in the mother and daughter bud cortices, while Bim1 was distributed only in the mother cell. Our simulation connotes that reduced Bim1-mediated bias on the tips of the growing MTs is not favorable for nuclear migration (**Fig 5D**). Our *in silico* study also predicts a differential activity of dynein in the mother and daughter cell cortex. While a strong dynein-pull on the cMTs from the mother cortex is not favorable for nuclear migration, an optimized dynein-pull from the daughter cortex transmitted via cMTs is essential for nuclear migration (**Fig 5E**). Together, our *in silico* model characterized that an effective cortical bias mediated by Bim1 and dynein in the mother cortex towards the daughter cell is essential for nuclear migration.

**Table 2:** Identification of key regulatory proteins involved in nuclear migration and kinetochore clustering in *C*. *neoformans*. *S*. *cerevisiae* genes involved in nuclear migration, microtubule function, and kinetochore clustering were enlisted along with their regulators. These genes were used to search the *C*. *neoformans* genome with tBLASTn at the FungiDB (http://fungidb.org/fungidb/). The source of *S*. *cerevisiae* gene products and their systematic names were obtained from the *Saccharomyces* genome database (http://www.yeastgenome.org/). *C*. *neoformans* gene numbers and their E-values were obtained from the FungiDB (http://fungidb.org/fungidb/) after the BLAST analysis. The function of each *S*. *cerevisiae* genes was obtained from the *Saccharomyces* genome database (http://www.yeastgenome.org/).

*S*. *cerevisiae* gene	Systematic name	*C*. *neoformans* gene	E-value	Function in *S*. *cerevisiae*
**KINESINS (per *S*. *cerevisiae*)**				
*CIN8*	YEL061C	-	No hits	Kinesin-5 (+end directed)
*KAR3*	YPR141C	CNAG_05752	1e-95	Kinesin (-end directed)
*KIP1*	YBL063W	CNAG_03453	7e-68	Kinesin-5 (+end directed)
*KIP2*	YPL155C	CNAG_07817 CNAG_06335	1e-58 1e-54	Centromeric protein E
*KIP3*	YGL216W	CNAG_00172	1e-95	Kinesin-related motor protein
*SMY1*	YKL079W	CNAG_06728	2e-42	Interacts with Myo2
**+TIPs**				
*BIK1*	YCL029C	CNAG_06352	1e-56	MT plus end associated protein
*BIM1/YEB1*	YER016W	CNAG_03993	3e-07	Generates an MT capture site at the cell cortex along with Kar9
*KAR9*	YPL269W	-	No hits	Nuclear migration
*PAC1*	YOR269W	CNAG_07440	2e-29	LIS1/NudF homolog, nuclear migration
**DYNEIN/DYNACTIN (per *S*. *cerevisiae*)**				
*DYN1/DHC1*	YKR054C	CNAG_05894	0.0	Heavy chain of cytoplasmic dynein
*PAC11*	YDR488C	CNAG_04407	2e-09	Dynein intermediate chain
*ACT5/ARP1*	YHR129C	-	-	Component of dynactin complex
*ARP10*	YDR106W	CNAG_04196	1e-04	Component of dynactin complex
*JNM1*	YMR294W	CNAG_03966	0.32	Component of dynactin complex
*NIP100*	YPL174C	-	No hits	Component of dynactin complex
**REGULATORS**				
*IPL1*	YPL209C	CNAG_01285	5e-95	Aurora kinase B
*SHE1*	YBL031W	-	No hits	Mitotic spindle protein, inhibits dynein function

Further, to validate the model’s prediction of Bim1’s function in imparting cortical bias on the tips of the cMTs penetrating the daughter buds, we studied the localization of Bim1 at different stages of the cell cycle in the wild-type cells. We functionally expressed Bim1-3xGFP under the native promoter in the strain CNNV119. Localization of Bim1 at the mother bud from the time of its emergence of the daughter bud possibly suggests the Bim1-mediated bias of the cMTs interacting with the mother cortex towards daughter bud (**Fig 5F**). Subsequently, we tested the model’s prediction of the differential activity of dynein in mother and daughter cells, by studying the localization of dynein during different stages of the cell cycle. We functionally expressed dynein-3xGFP under the native promoter in the strain CNNV116. Dynein puncta are found to be distributed between the mother and daughter cell during the cell cycle in a stage-specific manner (**Fig 5G**). In unbudded/small-budded cells, multiple dynein puncta are visible. As the cells proceed to the next stage of cell cycle, dynein possibly localizes to the tip of the migrating cMTs and gets localized to the spindle poles. Subsequently, dynein relocalizes to the cortex of both the mother and daughter cells (**Fig 5G)**. Clearly, there are many randomly distributed dynein puncta in the mother bud compared to the daughter bud (large condensed dynein puncta). The spatial distribution of dynein puncta across the whole mother bud and localized dynein puncta in the daughter bud of large-budded cells suggests differential activity/force transduction of dynein in the mother and daughter cell.

**Fig 6:**
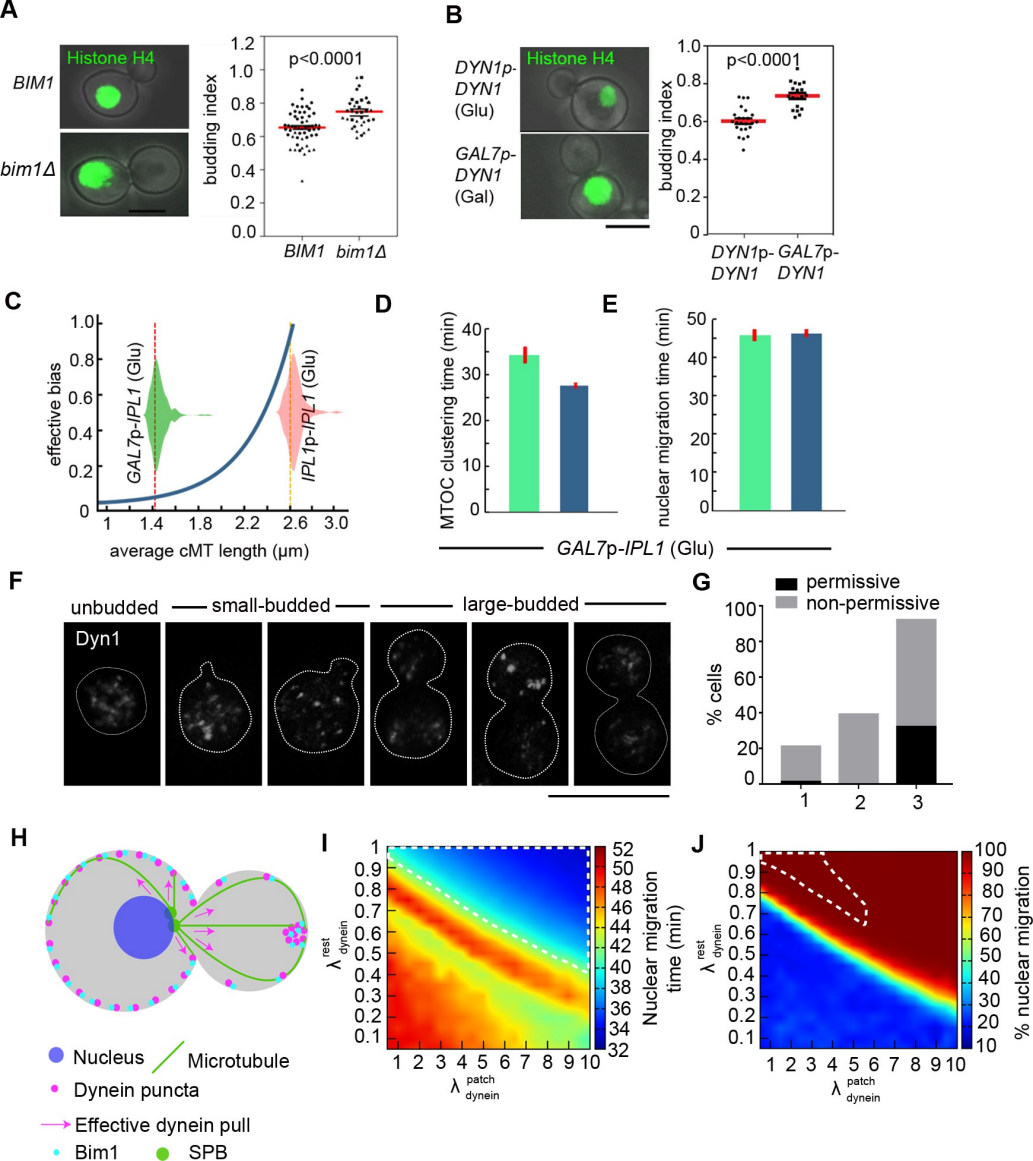
Ipl1-dependent microtubule integrity and Bim1-dynein-mediated effective bias ensure timely MTOC clustering and nuclear migration. (**A)** Analysis of budded cells with an unsegregated nucleus in the wild-type CNVY108 and *bim1*Δ CNNV107 cells expressing histone H4-GFP. Bar, 5μm (left). The cells with nucleus remained unsegregated in the mother bud or at the neck in the *bim1*Δ cells with the B.I. of 0.75 in contrast to 0.65 in the wild-type cells (n = 36) are plotted. Mean and SEM values are marked. p<0.0001, unpaired *t*-test (Right). (**B)** Defects in the nuclear migration in the wild-type CNVY108 and *DYN1*-overexpressing CNNV110 cells carrying histone H4-GFP. Bar. 5μm (left). The *DYN1-*overexpressing cells with nucleus remained unsegregated in the mother bud with the B.I. of 0.75 as compared to 0.6 in the wild-type cells. Mean and SEM values are marked. p<0.0001, unpaired *t*-test (Right). (**C)** The effective cortical bias increased exponentially with average cMT length (violin plots depict corresponding distributions) in the simulation. (**D-E)** The mean time required for the MTOC clustering and nuclear migration in the *ipl1* mutant cells (n≥32). (**F**) Localization of Dyn1 in the CNNV116 cells at different stages expressing Dyn1-3xGFP upon their growth in the non-permissive conditions. Bar, 10μm. (**G**) Quantification of aberrant spatial localization of dynein in the CNNV116 cells when grown in the permissive and non-permissive conditions. Percentages of cells having clustered dynein puncta in unbudded or budded cells (1), multiple dynein puncta in the daughter bud of large-budded cells (2) and no dynein puncta in the daughter bud of small-budded cells (3) are plotted, (n≥42). (**H**) In the schematic depicting the simulation framework, the dynein/ Bim1 puncta are distributed in the mother cortex (magenta dots) while in the daughter bud the dynein/ Bim1 puncta are condensed and localized. This spatial arrangement facilitates a net directed pull on the nucleus towards the daughter bud through the cMTs interacting with the daughter cortex. (**I**) The x-axis underlines the scale of dynein density in the condensed puncta within the daughter bud (which is $\lambda_{dynein}^{patch}$ times the constant dynein density *λ*_*dyn*_ = 6 μm^-1^ in the mother cortex). The y-axis represents the scale of dynein density outside the condensed patch in the daughter cortex ($\lambda_{dynein}^{rest}$ times the dynein density *λ*_*dyn*_ = 6 μm^-1^ in the mother cortex). The color map depicts the variation in nuclear migration time for different values of $\lambda_{dynein}^{patch}$ and $\lambda_{dynein}^{rest}$. The region demarcated by the white dashed border denotes the favorable parameter regime in $\lambda_{dynein}^{patch}‑\lambda_{dynein}^{rest}$ space where proper nuclear migration occurs within 35–40 min for ~100% cells. (**J**) Variation in the percentage of cells across a population of 1000 cells undergoing proper nuclear migration within a cut-off limit of 60 min. The region demarcated by the white dashed border denotes the favorable parameter regime in $\lambda_{dynein}^{patch}‑\lambda_{dynein}^{rest}$ space where not only proper nuclear migration occurs within 35–40 min for ~100% cells but additional spindle characteristics (e.g. spindle orientation, septin to spindle distance etc.) are also mapped reasonably well with the wild-type attributes observed in experiment.

Next, to test the model’s prediction of impaired nuclear migration due to a reduced bias and higher dynein activity in the mother cortex, we examined the dynamics of nuclear migration in the deletion mutant of *BIM1* CNNV107 (*Δbim1*) and a strain over-expressing *DYN1*, CNNV110 (*DYN1*^*OE*^). As predicted by the model, we observed that in the absence of Bim1 or in the presence of heightened dynein activity, the nuclear migration was delayed (**Fig 6A and 6B**). *In silico* model predicts that proper dynein activity is required for timely kinetochore clustering. The effect of the heightened and reduced activity of dynein on the dynamics of kinetochore clustering was tested in strain CNNV111 carrying GFP-CENP-A. Altered expression of dynein indeed leads to impaired dynamics of kinetochore clustering (**S4C, S4D, S4E and S4F Fig**). Taken together, *in silico* and experimental data, the synergistic presence of an effective bias due to the interplay between cortical determinants within the mother cortex and a more directed dynein-pull from the daughter cortex is indispensable for the proper nuclear division.

### Ipl1 regulates nuclear migration and kinetochore clustering by concomitantly altering microtubule dynamics and effective cortical bias in the mother bud

Experimental results suggested that the depletion of Ipl1 shortened the average cMT length from 2.63 μm to 1.3 μm, delayed kinetochore clustering time by 40% (10 min) and nuclear migration by 18% (12 min). Employing a characteristic mean cMT length (1.3 μm) in the model over non-identical sets of homogeneous cell population yields inconsistencies in the time required for nuclear migration and MTOC clustering (**S5A and S5B Fig**). The model predicted the time required for MTOC clustering and nuclear migration by introducing heterogeneous length of cMTs and perturbing the effective cortical bias mediated by Bim1 and dynein in the mother which also disagreed with the experiment. To account for the synergistic disruption of the cMT length and nuclear migration upon Ipl1 depletion in the model, we assumed that the level of effective cortical bias (a key factor for proper nuclear migration) diminishes exponentially with the average cMT length (**Fig 6C**). A combined effect of disintegrating MTs and diminishing bias in the mother cortex resulted in clustering of MTOCs in ~29 min and proper nuclear migration within ~47 min (**Fig 6D and 6E; S3B–S3D Movie**). In contrast to a uniform bias across population scenario (**S6C Fig**), *in silico* distribution for the neck to nucleus distance in presence of a length-dependent bias also closely emulated the experimental observation (**S6D Fig**). To test the model’s prediction of diminishing effective cortical bias in Ipl1-depleted cells, we first studied the localization of dynein in Ipl1-depleted cells of CNNV116 after their growth in non-permissive conditions for 8 h. We observed a reduction in the punctate localization of dynein in the Ipl1-depleted cells as compared to the cells grown in permissive conditions (**S6 Fig**). Upon increasing the exposure time using the definite intensity module, we observed aberrant spatial distribution of dynein puncta in Ipl1-depleted cells like clustered dynein puncta in unbudded or budded cells (20%), multiple dynein puncta in the daughter bud of large-budded cells (39%) or no dynein puncta in the daughter bud of small-budded cells (60%) (**Fig 6F and 6G**). Aberrant spatial distribution of dynein in the Ipl1-depleted cells possibly results in the net reduction in the directed dynein-mediated pull from the daughter bud required for proper nuclear migration. However, the decreased MT stability could also result in the aberrant localization of dynein puncta in the Ipl1-depleted cells.

To understand the significance of proper spatial distribution of dynein or Bim1 in nuclear migration, we introduced an additional refinement on the earlier *in silico* template having uniform dynein/Bim1 distribution in mother and daughter cortices (**Fig 6H**). We find that the higher Bim1 concentration in the mother bud imparts a greater bias to the cMTs interacting with the mother cortex, required for nuclear migration in to the daughter bud. Our model findings allude that there is a preferable range of effective dynein densities along the cortex resulting in timely nuclear migration (~35–40 min) across the overall population of cells (**Fig 6I**). In the configurations having the higher effective dynein density in the condensed patch in daughter cortex compared to the dynein density in the mother and elsewhere in the daughter cortex (demarcated in **Fig 6I)**, the randomly distributed dynein puncta in the mother cortex seem to lose the tug-of-war with respect to the focused dynein puncta in the daughter, facilitating timely nuclear migration in wild-type cells. Data obtained from the refined model shows that the parameter regime for proper nuclear migration becomes constrained when additional spindle characteristics are mapped with the experiments suggesting, apart from the puncta, uniformly distributed low-density dynein along the cortex might be crucial (**Fig 6I and 6J**). Further, we simulated the model considering the altered MT parameters and effective spatial distribution of dynein observed in Ipl1-depleted scenario. We find that a net reduction in the directed-pull on the nucleus from the daughter bud results in the delayed nuclear migration in the Ipl1-depleted cells as observed previously, when the uniform spatial profile of dynein and Bim1 was considered. Combining model refined predictions and experimental validations, we conclude that the differential spatial profile of dynein in the mother and daughter bud also orchestrates proper nuclear migration. Ipl1 spatio-temporally regulates cytoplasmic MTs and spatial distribution of Bim1 and/or dynein in the cell. Absence of Ipl1 impairs the effective cortical bias and the MT lengths leading to defects in kinetochore clustering and nuclear migration.

### Alterations in the dynamics of nuclear division in Ipl1-depleted cells cause aneuploidy and drug resistance

**Fig 7:**
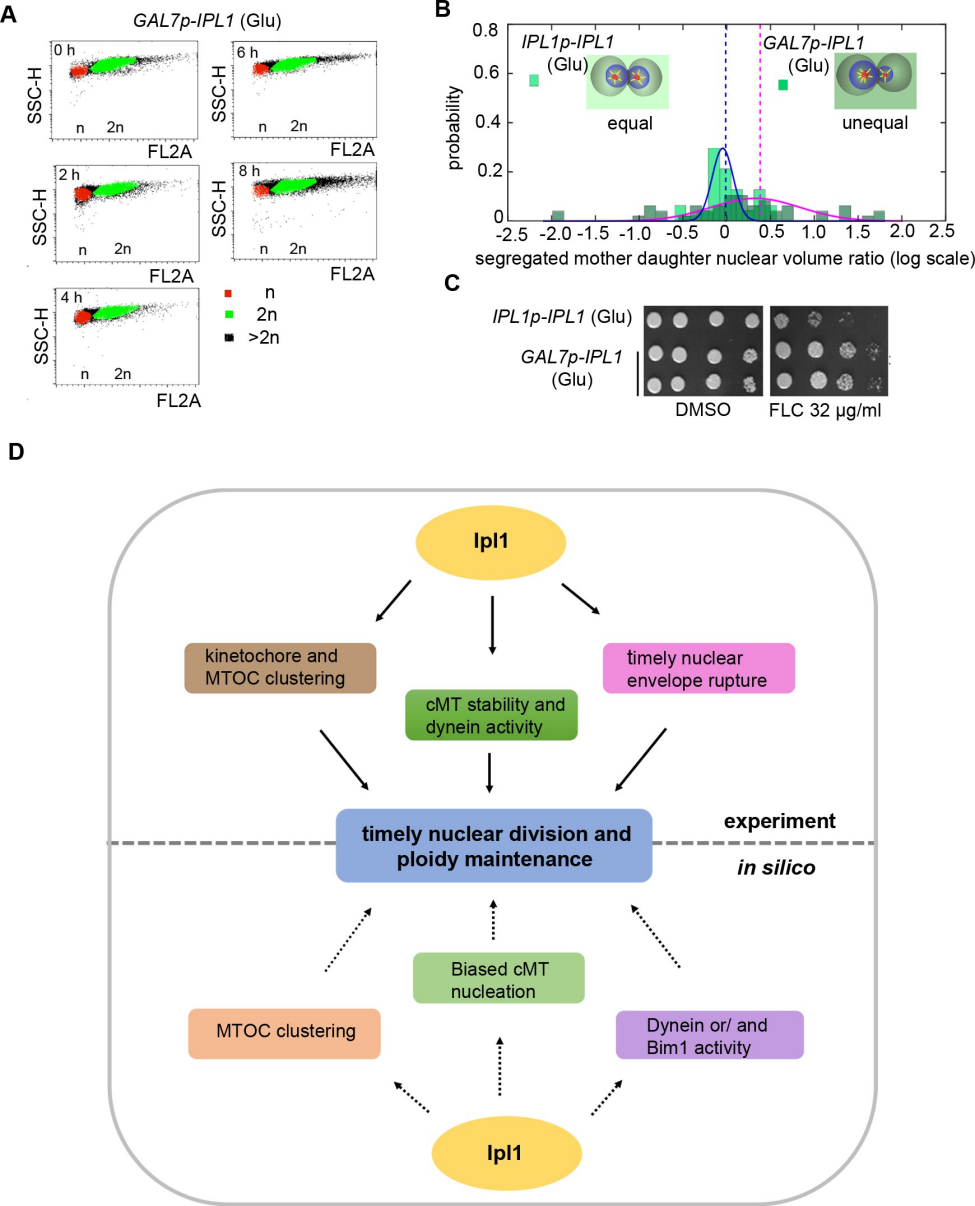
Ipl1 depletion induces aneuploidy and drug resistance in *C*. *neoformans*. **(A)** Dot plots depicting DNA content measured in the wild-type H99 and Ipl1-depleted CNNV101 cells by flow cytometry after growth in non-permissive conditions at the indicated time intervals. (**B)** Histograms of the segregated mother-daughter nuclear volume ratio in the wild-type and *ipl1* mutant cells in semi-log scale, portraying a symmetric distribution with mean around 0.0 for equal distribution. (**C)** Antifungal drug resistance assays corroborating aneuploidy in *GAL7p-IPL1* cells grown in non-permissive conditions for 8 h. Wild-type and Ipl1-depleted cells were 10-fold serially diluted (10^5^ to 10^2^) and spotted on the permissive medium containing an indicated concentration of fluconazole (FLC). (**D)** A model depicting cytosolic Ipl1 function in nuclear division. Ipl1 regulates cytoplasmic MT stability, kinetochore/MTOC clustering, dynein or/and Bim1 activity and nuclear envelope rupture to facilitate timely nuclear migration and ploidy maintenance in *C*. *neoformans*.

Next, we estimated the fidelity of chromosome segregation during mitosis in Ipl1-depleted cells due to altered nuclear migration and kinetochore clustering. The flow cytometric analysis (FACS) confirmed the existence of aneuploidy in a population of Ipl1-depleted cells within 8 h of protein depletion (**Fig 7A**). Volume rendering by Imaris 7.6.4 software (Bitplane, Zurich, Switzerland) reveals a biased distribution pattern of the chromatin mass in the Ipl1-depleted large budded cells. In most cases, a greater amount of the chromatin mass was present in the mother cell as compared to the daughter cell (**Fig 7B**). *C*. *neoformans* is known to acquire aneuploidy as an adaptive mechanism to exhibit heteroresistance against azole drugs [24]. Indeed, two independent *ipl1* conditional mutants CNNV101 and CNNV102, grown in the non-permissive condition for 8 h followed by growth on plates containing permissive media, yielded a higher number of fluconazole (32μg/ml) resistance colonies as compared to the wild-type (**Fig 7C**). Together, these results provide an evidence of the generation of aneuploidy upon depletion of Ipl1 in *C*. *neoformans*.

## Discussion

In this work, we show that Ipl1 is essential for viability and it orchestrates pre-anaphase nuclear movement from the mother cell to the daughter in the human pathogenic budding yeast *C*. *neoformans*. Ipl1 plays a critical role in maintaining MT stability to facilitate timely kinetochore clustering and nuclear migration. Our *in silico* study predicts that dynein and/or Bim1 activity regulates MTOC/kinetochore clustering, a pre-requisite for nuclear migration. By regulating the Bim1/dynein activity or localization, Ipl1 creates a net force bias necessary for proper nuclear migration to the daughter cell followed by its division during mitosis in *C*. *neoformans*. Cytosolic Ipl1 regulates these processes. It also regulates the disassembly of the NE to enter into the nucleus during mitosis and monitors the kinetochore-MT attachments to ensure high-fidelity chromosome segregation. Finally, we show that malfunctioning of Ipl1 can lead to aneuploidy-induced drug resistance in *C*. *neoformans*. Taken together, we demonstrate that dynamic localization of Ipl1 during semi-open mitosis spatio-temporally regulates the process of nuclear division and maintains proper ploidy in the human pathogen *C*. *neoformans* that belongs to the fungal phylum of Basidiomycota (**Fig 7D**).

In ascomyceteous yeast *S*. *cerevisiae*, Ipl1 displays a dynamic subcellular localization during the cell cycle [18]. In metaphase, Ipl1 associates with the centromeres and facilitates biorientation by preventing syntelic attachments [62]. In anaphase, Ipl1 localizes to the spindle midzone and regulates timely disassembly of the spindle followed by cytokinesis [9, 17, 18]. However, whether Ipl1 plays any role in regulating the dynamics of cMTs or associated cortical proteins remained unknown. In this study, we elucidated the role of Ipl1 in regulating cortical interactions in *C*. *neoformans* having atypical mitotic features [20]. A greater number of cMTs and an increased dynein activity at the daughter cortex in *C*. *neoformans* provide a stronger pulling force on the migrating nucleus as compared to the *S*. *cerevisiae* [19]. Biased polarisation of cMTs towards the daughter cell and asymmetric recruitment of accessory cytoplasmic proteins such as Myo2, Bim1, Kar9, dynein etc. have been shown to influence nuclear migration [8, 19, 59, 60]. In the absence of a functional homolog of Kar9 [61], which imparts a cortical bias on the cMT tips penetrated into the cortex [60] and She1 which inhibits dynein activity along the MTs in *S*. *cerevisiae* [8], it is conceivable that an altered mechanism is operational to drive the process of nuclear migration in *C*. *neoformans*. Owing to significant differences in the dynamics of nuclear migration and kinetochore clustering as compared to well-studied yeast species, and the absence of Kar9 and She1, *C*. *neoformans* provides a unique system to study the evolution of chromosome segregation across budding yeast species.

Majority of Ipl1-depleted cells in *C*. *neoformans* exhibit defects in nuclear migration in contrast to most *ipl1-2* cells with unequally segregated chromosomes in *S*. *cerevisiae* [32]. Kinetochores are closely associated with MTOCs (and SPBs) throughout the cell cycle in *C*. *neoformans*. Ipl1-depleted cells exhibit altered MTOC clustering dynamics which in turn possibly results in delayed kinetochore clustering. The compromised structural stability of MTs suggests that cytoplasmic Ipl1 possibly functions either as an MT stabilizer or as a negative regulator of MT-severing enzymes and regulate nuclear migration in *C*. *neoformans*. It is also possible that gross MT abnormalities and formation of “tubulin” blobs in Ipl1-depleted cells could be an indirect consequence of Ipl1 depletion through the altered activity of MAPs or kinesin or improper tubulin folding. A series of evidence indicated that motor proteins such as the kinesin-8 homolog, Kip3 is involved in kinetochore clustering in *S*. *cerevisiae* [63], but its function remains uncharacterized in *C*. *neoformans*. While MTs are stabilized by end-binding proteins such as Bim1 [60], Ipl1 triggers spindle disassembly by phosphorylating Bim1 in *S*. *cerevisiae* [9, 17]. Recently, it has been shown that Ipl1 inhibits spindle stabilization by negatively regulating the MT-stabilizing protein She1 in *S*. *cerevisiae* [16]. Using spatio-temporal simulations, we find that pathways involving cortical bias triggered by Bim1 and cytoplasmic dynein facilitate nuclear migration and MTOC clustering in *C*. *neoformans*.

A constitutively high activity of Bim1 and/or dynein throughout the cell cycle results in defective spindle disassembly and nuclear migration [16, 17, 64]. In *C*. *neoformans*, dynein/Bim1 puncta are distributed in the mother cortex while these puncta are condensed and localized in the daughter bud. Since the spatial profile of the dynein in the mother cell is less condensed and distributed, the resultant opposing pull on the nucleus in the mother cell is substantially reduced, hence lacks in competition against the pull from the daughter for proper migration. Furthermore, the net pull from the mother bud is also suppressed by the enriched spatial profile of Bim1 in the mother cortex contributing to a net effective bias of the pulling force on the nucleus towards the daughter bud. Thus, this spatial arrangement facilitates a net directed-pull on the nucleus towards the daughter bud through the cMTs interacting with the daughter cortex. The spatial distribution of dynein puncta in the mother and daughter bud is found to be significantly altered in Ipl1-depleted cells. Our model findings subscribe that for a suitable range of effective dynein densities along the cortex and the higher effective dynein density in the condensed patch in daughter cortex is important for proper nuclear migration. It is possible that Ipl1 regulates the timely nuclear migration by maintaining the effective spatial distribution profile of dynein/Bim1 in the mother and daughter cell. Irrespective of whether the *in silico* structural template contains uniform dynein distribution at the mother and the daughter cortex or punctated distribution, our model findings consistently refer to the importance of stronger dynein pull from the daughter bud compared to the mother bud in orchestrating proper nuclear migration.

Presence of cytosolic Ipl1 throughout the cell cycle indicates that Ipl1 is involved in regulation of the activity of cytosolic proteins in *C*. *neoformans*. Indeed, a varying cytosolic to the nuclear pool of Ipl1 across various stages may explain a cell cycle-dependent differential regulation of Bim1 and/or dynein in *C*. *neoformans*. In the absence of the direct experimental evidence on how Ipl1 alters the characteristics of dynein/Bim1/MTs in *C*. *neoformans*, we used an *in silico* approach and reproduced the Ipl1-depleted phenotypes observed in the experiments. The purpose of the model is to predict plausible mechanism(s) by which the nuclear migration and kinetochore clustering is delayed in Ipl1-depleted cell. Assuming Ipl1 depletion alters effective dynein/Bim1-mediated bias and enhances MT catastrophe, we could reproduce the mutant phenotypes observed in the experiment using our simulated model. Applying perturbations in dynein/Bim1 profiles and densities along with variations in MT stability as observed in the experiment, we could successfully capture the semi-quantitative characteristics of spindle architecture in both wild-type and Ipl1-depleted cells. Although, the simplified *in silico* framework provides a plausible scenario of the underlying molecular interactions, the model has certain limitations. Specifically, the model neglects considering explicit mechanism how dyneins/Bim1 stochastically attaches and detaches with MTs at the cortex and move along the MT filament toward the SPBs. The model further ignores the dynamic clustering of dyneins that might spatio-temporally regulate pulling on the nucleus via MTs along the course of nuclear migration. Such limitations of a coarse-grained approach invite a more refined approach to be addressed in the future.

The depletion of Ipl1 using the well characterized *GAL7* promoter system over a period of many cell cycles in an unsynchronized cell culture is likely to create heterogenous phenotypes of defective kinetochore clustering, nuclear migration and microtubule length in *C*. *neoformans*. Alternatively, this effect could be an indirect consequence of chromosome mis-segregation in the previous cell cycles. Recently, condensin has been shown to play an indirect role in the regulation of the gene expression in fission yeast [65]. It is important for the maintenance of proper gene expression by regulating accurate chromosome segregation during mitosis [65]. Therefore, development of a single cell cycle depletion assay such as with cell cycle arrest and release or auxin-based depletion system would be more useful to rule out the indirect consequences of depletion of Ipl1 in *C*. *neoformans*. In this study, we propose that semi-open mitosis maintains Ipl1’s function in time and space by regulating the timely entry of Ipl1 into the nucleus that is allowed only when the NE ruptures at during mitosis in *C*. *neoformans*. Taken together, we posit that cytosolic pool of Ipl1 regulates the overall MT integrity, and Bim1 or/ and dynein activity to ensure high-fidelity chromosome segregation and prevent aneuploidy-associated drug resistance in *C*. *neoformans*.

## Materials and methods

### Yeast strains, plasmids, and media conditions

Strains and primers used in this study are listed in the **S1 Table** and **S2 Table** respectively.

### Construction of the conditional mutant of *IPL1*

The conditional mutant strains of *IPL1* were constructed by replacing the promoter of the *IPL1* ORF (CNAG_01285) with the *GAL7* promoter [33]. The 5’ coding region of the *IPL1* ORF was amplified from the H99 genomic DNA using oligos listed in the **S2 Table** and cloned into KpnI and XhoI sites of the plasmid pSHG7 that contained the *GAL7* promoter and the hygromycin (*HygB*) marker, to construct pG7IPL1. The 5’ untranslated region (UTR) of the *IPL1* ORF was amplified from the H99 genomic DNA using oligos listed in the **S2 Table** and cloned into the NotI site of pG7IPL1 to obtain pUSG7IPL1. After confirmation of the orientation of the insert, the resulting plasmid was used to amplify the entire deletion cassette using the primers (NV123 and NV118) and introduced into each of H99 [66], CNVY107 and CNVY108, using biolistic transformation [67] to obtain CNNV101, CNNV102, CNNV105, and CNNV104 respectively. All the conditional mutants were confirmed by PCR using oligos listed in the **S2 Table**.

To visualize the signals of outer kinetochore proteins such as Mtw1 and Dad1 under Ipl1-depleted conditions, we constructed conditional *ipl1* mutant strains in CNVY103 and CNVY120 strain backgrounds. The cassette containing the upstream homology region from *IPL1*, the *GAL7* promoter, *HygB* and the 5’ UTR of the ORF was amplified using primers mentioned above and transformed into the Mtw1- and Dad1- epitope-tagged strains to obtain CNNV108 and CNNV109 respectively. These conditional mutants were confirmed by their inability to grow under non-permissive media conditions.

### Construction of a strain expressing mCherry-tagged Ipl1 under the *GAL7* promoter

To study the dynamic subcellular localization of Ipl1, we constructed the strain expressing Ipl1 tagged with mCherry under the *GAL7* promoter (CNNV114). The mCherry coding DNA sequence was amplified from the plasmid pS2M using oligos listed in the **S2 Table** and cloned into BamHI and XhoI sites of the plasmid pUSG7IPL1 that contained the 5’ coding region of the *IPL1* ORF, 5’ UTR of the *IPL1* ORF, *GAL7* promoter, and *HygB* marker, to construct pUSG7mchIPL1. The resulting plasmid was used to amplify the entire tagging cassette using the primers (NV132 and NV118) and introduced into H99 and CNVY108 using biolistic transformation [67] to obtain CNNV103 and CNNV114 respectively.

To the distinguish the nuclear localization of Ipl1 from that of cytosol, we constructed the strain co-expressing proliferating cell nuclear antigen (PCNA) tagged with GFP and Ipl1 tagged with mCherry under the *GAL7* promoter (CNNV112). The 5’ coding region of the *PCNA* ORF (CNAG_06079) was amplified from the H99 genomic DNA using oligos listed in the **S2 Table** and cloned into the BamHI site of the plasmid pCIN19 that contained the GFP and the nourseothricin (*NAT)* marker. The resulting plasmid was transformed into CNNV103 to obtain CNNV112.

### Construction of a strain expressing triple GFP epitope-tagged Ipl1 under the native promoter

To study the subcellular localization of Ipl1 under the native promoter, we constructed the strain expressing Ipl1 tagged with triple GFP under the native promoter (CNNV113). The 5’ coding region of the *IPL1* ORF was amplified from the H99 genomic DNA using oligos listed in the **S2 Table** and cloned into ClaI and XhoI sites of the plasmid pB1586 that contained the triple GFP epitope, to construct pIPL1GFP3. The 3’ untranslated region (UTR) of the *IPL1* ORF was amplified from the H99 genomic DNA using oligos listed in the **S2 Table** and cloned into the SacII site of pIPL1GFP to obtain pUSIPL1GFP3. After confirmation of the orientation of the insert, the resulting plasmid was used to clone the neomycin (*NEO*) resistance gene. A fragment (2 kb) containing *NEO* was amplified from pLK25 [20] using oligos listed in the **S2 Table** and cloned into the NotI site of pUSIPL1GFP3, to obtain pUSIPL1GFP3N. The resulting plasmid was digested with XhoI and NdeI and introduced into H99 using biolistic transformation [67] to obtain CNNV113.

### Construction of the deletion mutant of *BIM1* expressing GFP-tagged histone H4

To visualize the nucleus by localizing histone H4 in the absence of Bim1, we constructed the *bim1*Δ strain in a GFP-H4-expressing strain (CNVY107) background. The *BIM1* ORF (CNAG_03993) was replaced with the neomycin (*NEO*) resistance gene cassette to generate *bim1*Δ mutants. The *BIM1* knockout cassette containing *NEO* was generated by the overlap PCR strategy described earlier [20] using oligos listed in the **S2 Table**. Approximately 1 kb each of 3’and 5’ UTR of the gene was amplified from the H99 genomic DNA. A fragment (2 kb) containing *NEO* was amplified from pLK25 [20]. All the three fragments were mixed to perform overlap PCR for generating the *BIM1* deletion cassette. The amplified *bim1*Δ: *NEO*^R^ cassette was introduced into CNVY108 (expressing GFP-H4) using biolistic transformation [67] to obtain CNNV107.

### Construction of the conditional mutant of *DYN1* expressing GFP-tagged CENP-A and GFP-tagged H4

The conditional mutant strains of *DYN1* were constructed by replacing the promoter of the *DYN1* ORF (CNAG_05894) with the *GAL7* promoter [33]. The 5’ coding region of the *DYN1* ORF was amplified from the H99 genomic DNA using oligos listed in the **S2 Table** and cloned into BamHI and SalI sites of the plasmid pSHG7 carrying the *GAL7* promoter and *HygB* to construct pG7DYN1. The *DYN1* 5’UTR was amplified from H99 using oligos listed in the **S2 Table** and cloned into the NotI site of pG7DYN1 to obtain pUSG7DYN1. After confirmation of the orientation of the insert, the resulting plasmid was used to amplify the cassette using the primers (NV404 and NV356) and introduced into each of H99 [66], CNVY108 and CNVY113 strains, using biolistic transformation [67] to obtain CNNV110 and CNNV111 respectively. All the conditional mutants were confirmed by PCR using oligos listed in the **S2 Table**.

### Construction of a strain expressing triple GFP epitope-tagged Dyn1 under the native promoter

To study the subcellular localization of Dyn1 under the native promoter, we constructed the strain expressing Dyn1 tagged with triple GFP under the native promoter (CNNV116). First, a fragment (2 kb) containing *NEO* was amplified from pLK25 [20] using oligos listed in the **S2 Table** and cloned into the NotI site of pIPLGFP3, to obtain pCI3GFPN. The 5’coding region of the *DYN1* ORF was amplified from the H99 genomic DNA using oligos listed in the **S2 Table** and cloned into ClaI and KpnI sites of the plasmid pCI3GFPN that contained the triple GFP epitope and *NEO* resistance gene, to construct pCD3GFPN. The 3’untranslated region (UTR) of the *DYN1* ORF was amplified from the H99 genomic DNA using oligos listed in the **S2 Table** and cloned into the SacI site of pCD3GFPN to obtain pCD3GFPNDS. After confirmation of the orientation of the insert, the resulting plasmid was used as a template to amplify DYN1-3xGFP DS cassette using oligos NV452 and NV455 and introduced into CNNV101 using biolistic transformation [67] CNNV116.

### Construction of a strain expressing triple GFP epitope-tagged Spc98 under the native promoter

To study the subcellular localization of MTOCs under the native promoter, we constructed the strain expressing Spc98 tagged with triple GFP under the native promoter (CNNV118). The 5’ coding region of the *SPC98* ORF was amplified from the H99 genomic DNA using oligos listed in the **S2 Table** and cloned into ClaI and KpnI sites of the plasmid pCI3GFPN that contained the triple GFP epitope and *NEO* resistance gene, to construct pCS3GFPN. The 3’ untranslated region (UTR) of the *SPC98* ORF was amplified from the H99 genomic DNA using oligos listed in the **S2 Table** and cloned into the SacI site of pCS3GFPN to obtain pCS3GFPNDS. After confirmation of the orientation of the insert, the resulting plasmid was used as a template to amplify SPC98-3xGFP DS cassette using oligos, NV478 and NV477 and introduced into CNNV101 using biolistic transformation [67] to obtain CNNV118.

### Construction of a strain expressing triple GFP epitope-tagged Bim1 under the native promoter

To study the subcellular localization of Bim1 under the native promoter, we constructed the strain expressing Bim1 tagged with triple GFP under the native promoter (CNNV119). The 5’ coding region of the *BIM1* ORF was amplified from the H99 genomic DNA using oligos listed in the **S2 Table** and cloned into ClaI and KpnI sites of the plasmid pCI3GFPN that contained the triple GFP epitope and *NEO* resistance gene, to construct pCB3GFPN. The 3’ untranslated region (UTR) of the *BIM1* ORF was amplified from the H99 genomic DNA using oligos listed in the **S2 Table** and cloned into the SacI site of pCB3GFPN to obtain pCB3GFPNDS. After confirmation of the orientation of the insert, the resulting plasmid was used as a template to amplify BIM1-3xGFP DS cassette using oligos, NV470 and NV473 and introduced into H99 using biolistic transformation [67] to obtain CNNV119.

### Media and growth conditions

The conditional mutant strains CNNV101, CNNV102, CNNV110 and CNNV111 carrying *IPL1* and *DYN1* respectively, under the control of the *GAL7* promoter were grown in YPG (1% yeast extract, 2% peptone, 2% galactose) as a permissive medium and YPD (1% yeast extract, 2% peptone, 2% dextrose) as a non-permissive medium. The deletion mutant strains were grown in YPD. All the *C*. *neoformans* strains were grown at 30°C.

### Live-cell imaging

The conditional mutant strains, CNNV104 and CNNV105, were grown overnight in the permissive medium and re-inoculated in the non-permissive media for 4 h. The deletion mutant strain, CNNV107 was grown in YPD overnight. These cells were pelleted at 4,000 rpm and washed once with 1x phosphate buffered saline (PBS). The cell suspension was placed on the slide containing a thin 2% agarose patch prepared in dextrose and the patch was covered with a coverslip. Live-cell imaging was performed at 30°C on an inverted confocal microscope (ZEISS, LSM-880) equipped with a temperature-control chamber (Pecon incubator, XL multi SL), a Plan Apochromat 100x NA oil 1.4 objective and GaAsp photodetectors. Two-color images were taken by the sequential switching between RFP and GFP filter lines (GFP/FITC 488, mCherry 561 for excitation and GFP/FITC 500/550 band-pass, mCherry 565/650 long-pass for emission). For time-lapse microscopy of mCherry-CENP-A, histone H4-GFP, and GFP-Tub1, images were collected at a 2-min interval with 1.5% intensity exposure, a 60-s interval with 0.2% intensity exposure and a 60-s interval with 1% intensity exposure respectively, with 0.5 μm Z-steps. All the images were displayed after the maximum intensity projection of images at each time using ImageJ.

### Microscopic image acquisition and processing

The conditional mutant strains, CNNV104, CNNV105, CNNV108, CNNV109, and CNNV116 were grown till OD_600_ = 1 in the permissive medium and re-inoculated in the non-permissive media for 8 h. The deletion mutant and CNNV113 strains were grown in YPD overnight. These cells were pelleted at 4,000 rpm and washed once with 1x phosphate buffered saline (PBS) before the cell suspension was placed on a thin growth medium containing 2% agarose patch present on the slide. A coverslip was placed on the patch and processed for imaging. The images were acquired at room temperature using laser scanning inverted confocal microscope LSM 880-Airyscan (ZEISS, Plan Apochromat 63x, NA oil 1.4) equipped with highly sensitive photo-detectors or the DeltaVision System (Applied Precision) or Axio Observer Calibri (ZEISS). The filters used were GFP/FITC 488, mCherry 561 for excitation and GFP/FITC 500/550 band pass, mCherry 565/650 long pass for emission. Z- stack images were taken at every 0.3 μm and processed using ZEISS Zen software/ImageJ. The Airyscan images were subjected to the super-resolution mode processing using ZEISS zen software. All the images were digitally altered with minimal adjustments to levels and linear contrast till the signals were highlighted.

### Post-acquisition analysis

The length of an MT fiber was measured by tracking it manually using a freehand line tool in ImageJ. The end-to-end distance was measured for the intact MTs while the longest possible distance along the clustered MT structure was measured for the disintegrated MTs. The nuclear volumes were measured after the 3D rendering of confocal images with IMARIS 7.6.4 software (Bitplane, Zurich, Switzerland). Images were filtered by Gaussian smoothing and a surface was created using a threshold of absolute intensity. Radii of the nuclei spots were determined by taking a half of the longest diameters for each nucleus spot measured in an individual stack in Imaris. The nuclear volumes were obtained in statistics of processed images and these values were used to calculate nuclear volume ratio of the mother versus daughter cell.

### Immunoblotting

Wild-type and mutant cells were grown under permissive and non-permissive conditions for the indicated time points. The cells of OD_600_ = 3 were harvested and washed with 1x PBS. The cells were resuspended in 12.5% TCA and disrupted using acid-washed glass beads (Sigma, Cat. No. G8772) by vortexing for 30 min at 4°C. Cell lysates were precipitated at 13000 rpm for 10 min and washed with 80% acetone. The pellet was dried and resuspended in the lysis buffer (0.1 N NaOH, 1% SDS). The samples were diluted in 5x SDS loading dye (5% β-mercaptoethanol, 0.02% Bromophenol blue, 30% glycerol, 10% SDS, 250 mM Tris-Cl pH 6.8) and denatured. Denatured samples were subjected to electrophoresis using 10% SDS PAGE and transferred to a nitrocellulose membrane for 45 min at 25 V by semi-dry method (Bio-Rad). The membranes were blocked with 5% skim milk containing 1x PBS (pH 7.4) for 1 h at the room temperature followed by its incubation with primary antibodies in 2.5% skim milk overnight at 4°C. After three 10 min washes in PBST (1x PBS, 0.05% Tween) solution, the membranes were incubated in solutions containing secondary antibodies in 2.5% skim milk for 2 h. The membranes were washed with PBST (1X PBS, 0.05% Tween) solution thrice and the signals were detected using chemiluminescence method (SuperSignal West Pico Chemiluminescent substrate, Thermo Scientific, Cat. No. 34080).

### Antibodies

Primary antibodies used for western blot analysis were mouse anti-GFP (dilution 1:2500) (Santa Cruz Biotech, Cat. No. 9996) and mouse anti-PSTAIRE (dilution 1:2000) (Abcam, Cat. No.10345). Secondary antibodies used are goat anti-mouse HRP conjugated antibodies (dilution 1:10,000) (Bangalore Genei, Cat. No. HP06).

### Anti-fungal drug resistance assay

The wild-type and Ipl1-depleted cells grown in the non-permissive condition for 8 h were 10-fold serially diluted (10^5^ to 10^2^) and spotted on plates containing the permissive medium having indicated concentrations of fluconazole (Sigma, Cat. No. F8929-100 MG) and incubated further at 30°C for the antifungal drug resistance assay.

### Statistical analyses

The values of the MT length, budding indices, mother/daughter nuclear volume ratios, kinetochore clustering and nuclei migration time durations were plotted as violin plots overlaid with the column scatter graph in a column type mean with the SEM using the Prism 7 software. Statistical differences were determined using a paired Student’s *t*-test to calculate the statistical significance. In all the *in silico* results reported, the quantification of various attributes were averaged over 2000 samples and plotted as column type means with SEM.

### Spatio-temporal simulation of kinetochore (KT) clustering, nuclear migration, and KT-MT interaction

#### Generation of the computational model

In this section, we give a detailed account of the key components of the coarse-grained 3-dimensional model and stochastic dynamics of various ingredients (see **Table 1**) to explore relevant aspects of mitotic division in *C*. *neoformans*.

#### Designing the mother cell and the daughter bud

The mother cell was modeled as a sphere of radius *r*_mother._ The daughter bud was initiated at a random position on the cell surface and its growth was calibrated in concert with the experiment. Using budding index (B.I.), defined as the ratio between the final diameter of the daughter bud and the mother bud as a reference, various mitotic events were addressed in a sequential manner. The daughter bud ceased to grow after the final budding index obtained from experiment [19].

#### Modeling of the nucleus and MTOCs

We considered the nucleus as a sphere of radius *r*_nuc_ placed at a random position within the mother cell at the onset of the simulation. MTOCs were also modeled as spheres embedded on the nuclear envelope (NE) and distributed randomly [38, 49].

#### Modeling cytoplasmic MTs and interaction with the cell cortex

Each cytoplasmic MT (cMT) filament was modeled as a rod of zero thickness undergoing dynamic instability, governed by characteristic instability parameters and the geometry of the confinement. The plus end of the cMT was considered to grow and shrink with velocity *v*_g_ and *v*_s_ respectively. The stochastic transition from the growing state to the shrinking state occurs when cMTs undergo catastrophe and was determined by frequency *f*_c_ [68]. Similarly, the transition rate between the shrinking to the growing state occurs when MTs undergo rescue and was determined by frequency *f*_r_ [68, 69]. These four independent parameters *v*_g,_ *v*_s,_ *f*_c_ and *f*_r,_ can mimic the MT dynamics at a relevant resolution. We assumed the cell cortex as a steric barrier of finite width *w*_cor_ along the cell periphery that impedes the unconstrained cMT growth resulting in a decrease in *v*_g_ within the cortical region. Owing to the impedance encountered at the cortex, cMTs experience an elastic repulsive force *f*_*push*_ *= l*_*cor*_*K*_*cor*_, during their growth phase inside the cortex. Here, *l*_*cor*_ depicts the penetration length of the cMT into the cortex and *K*_*cor*_ stands for the average stiffness of the cortical network. Within the cortex, the mechanical pull on the cMT due to collective activity of dynein motors is *f*_*pull*_ *= l*_*cor*_*λ*_*dyn*_*f*_*dyn*_, where *λ*_*dyn*_ is the linear density of dynein motors across the cMT segment passed into the cortex and *f*_*dyn*_ is the pulling force exerted by a single dynein [19]. Upon hitting the cell boundary, the growing cMTs either buckle along the cortex or switch to the depolymerization state. The cMTs that start to shrink upon hitting the cell boundary experience a pushing force impulse (*f*_*wall*_) ~1 pN [19]. In the buckling scenario, the tips of the cMTs are anchored to the cell-boundary and the cMTs start to bend producing the first order buckling force *f*_*buckling*_ *~1/l*^*2*^, where *l* is the total cMT length. cMTs slide along the cortex when the angle of incidence with respect to the cortex is small.

The net load force exerted on the cMT due to cortical impedance alters the overall dynamics of the cMT. Henceforth, the cMT parameters *v*_g_ and *f*_c_ are modified following. I) *v*_*g*_ *= v*_*g0*_*exp(f*_*load*_*/f*_*stall*_*)*, where *v*_*g0*_ is the unconstrained growth velocity when no load is applied to the cMTs, *f*_*load*_ is the magnitude of the load force and *f*_*stall*_ is the stall force per cMT. II) *f*_*c*_ *= f*_*c*_^*stall*^*/*[*1+*(*f*_*c*_^*stall*^*/f*_*c0*_*-1)*exp*(f*_*load*_*/f*_*stall*_*)*], where *f*_*c*_^*stall*^ is the rate of catastrophe of a stalled cMT and *f*_*c0*_ is the catastrophe rate of a free cMT [51].

In *C*. *neoformans*, the cortical interaction with bias mechanism is activated following simultaneous accumulation of Bim1 (generate biased movement of the cMT plus end) and dynein at the plus-end of the cMTs passed through the cortical region of mother bud. Following SPB duplication, cortical interaction with bias is implemented on the cMTs nucleated from the mother SPB. The force due to Bim1 is directed towards the septin ring and assumed to follow a similar characteristic that of a dynein. The collective activity of cortical determinants including Bim1 and dynein result in an effective pull on the cMT towards the septin ring, which subsequently acts on the nucleus. Long sliding cMTs often pass through the mother-daughter juncture and penetrate into the daughter cortex. These cMTs experience strong effective dynein pull originated at daughter cortex thus dragging the nucleus towards the daughter.

#### Modeling nuclear MTs, kinetochores and KT-kMT interaction

During mitosis, two different subsets of nuclear MTs (nMTs) i.e. ipMTs and kMTs are present inside the nucleus. Clustered kinetochores are attached to SPBs via the kMTs. The cross-linking and subsequent activities of kinesin-5 motors at the ipMT overlap [70] results in a force *f*_*ipMT*_ *= l*_*overlap*_*λ*_*ipMT*_*f*_*kinesin-5*_, where *l*_*overlap*_ is the total overlapping length among all the ipMTs nucleated from the duplicated SPBs, *λ*_*ipMT*_ is the linear density of kinesin- 5 motors at the overlap and *f*_*kinesin-5*_ is the force triggered by a single kinesin-5 motor [19]. The kinetochore is modeled as a sphere immersed in the viscous nucleoplasm having the coefficient of viscosity *η*_*np*_.

The kMTs were modeled in a similar fashion as the cMTs; though having a different set of dynamic instability parameters due to additional constraints imposed by the kinetochore geometry. Dynamic instability of the kMTs causes the kinetochores to experience a myriad of competing forces [71–73]. Throughout the polymerization phase, the growing kMT plus tip exerts a pushing force *f*_*poly*_ *= l*_*pen*_*K*_*fibril*_, where *l*_*pen*_ is the penetration length of the kMT inside the kinetochore and *K*_*fibril*_ is the stiffness constant of the kinetochore fibril spring. In the depolymerization phase, the shrinking kMT plus tip pulls the kinetochore with a force *f*_*depoly*_ *= l*_*gap*_*K*_*c*_, where *l*_*gap*_ is the distance of the gap between the kMT plus tip and the kinetochore and *K*_c_ stands for the spring constant of the spring connecting the kMT tip to the kinetochore [74].

In order to avoid overlap between two kinetochores, a hard-core repulsion is introduced amongst the kinetochores. If any two kinetochore spheres overlap, the corresponding repulsive force is considered as *f*_*intersection*_ *= Cd*_*overlap*_, where *d*_*overlap*_ is the finite overlap between two kinetochore spheres and *C* is the constant for the repulsion strength between two kinetochores which is taken to be 1pN/μm [19].

A pair of sister kinetochores remained attached to each other by the cohesin spring until metaphase. The cohesin spring introduces a Hookean force *f*_*cohesin*_ *= K*_*cohesin*_*d*_*separation*_ between the sister kinetochores where *K*_*cohesin*_ is the spring constant of the cohesin spring and *d*_*separation*_ is the separation distance between the sister kinetochores [19].

In order for the maintenance of the constant separation between the SPB and the kinetochore cluster and regulation of the spindle assembly, an additional mechanism is required to control kMT length. To implement this in the mechanistic model, the length-dependent catastrophe of kMTs [19, 57], is taken into account where the catastrophe frequency of a kMT increases linearly with the length *l*_kMT_ as *f*_c_ = *hl*_kMT._

#### Equations of motion for the nucleus, MTOCs, SPB, and kinetochore

Forces due to a single cMT (*f*_*push*_, *f*_*pull*_, *f*_*wall*_, *f*_*buckling*_) act on the nucleus and the MTOCs/SPBs, simultaneously. In addition, both mother and daughter SPBs experience ipMTs mediated repulsion (*f*_*ipMT*_) concurrently. If ${\vec{F}}_{nucleus}$, ${\vec{F}}_{MTOC}$ and ${\vec{F}}_{SPB}$ are the resultant forces acting on the nucleus, MTOC and the SPB, respectively, the corresponding equations of motion can be written as

$$\frac{d{\vec{X}}_{\mathrm{nucleus}}}{dt}=\frac{{\vec{F}}_{\mathrm{nucleus}}}{\xi_{\mathrm{nucleus}}}$$ (1)

$$\frac{d{\vec{X}}_{MTOC}}{dt}=\frac{{\vec{F}}_{MTOC}}{\xi_{MTOC}}$$ (2)

$$\frac{d{\vec{X}}_{SPB}}{dt}=\frac{{\vec{F}}_{SPB}}{\xi_{SPB}}$$ (3)

where, ${\vec{X}}_{nucleus}$, ${\vec{X}}_{MTOC}$, ${\vec{X}}_{SPB}$ are the instantaneous positions of the nucleus, MTOC and the SPB, respectively while *ξ*_*nucleus*_, *ξ*_*MTOC*_, *ξ*_*SPB*_ are corresponding viscous drags. Here, ${\vec{F}}_{nucleus}$, ${\vec{F}}_{MTOC}$ and ${\vec{F}}_{SPB}$ are all different functions of *f*_*push*_, *f*_*pull*_, *f*_*wall*_, *f*_*buckling*_, *f*_*ipMT*_. The above equations manifest the well-known Stokes law, $\vec{v}=\vec{F}/\xi$, in a viscous medium where, $\vec{v}$, $\vec{F}$ and ξ are the velocity, force and the viscous drag of a moving particle, respectively. Similarly, the motion of a single kinetochore is governed by the following equation of motion

$$\frac{d{\vec{X}}_{KT}}{dt}=\frac{{\vec{F}}_{KT}}{\xi_{KT}}$$ (4)

Here, ${\vec{X}}_{KT}$, ${\vec{F}}_{KT}$ and *ξ*_*KT*_ are the instantaneous position, resultant force and viscous drag experienced by kinetochore. Here ${\vec{F}}_{KT}$ is a function of *f*_*poly*_, *f*_*depoly*_, *f*_*intersection*_, *f*_*cohesin*_. The equations of motion are solved using Euler’s method.

## Supporting information

S1 Fig Sequence conservation and domain analysis of Aurora kinases. **A.** Phylogenetic analysis of Aurora kinases. Amino acid sequences for Aurora kinases in *H*. *sapiens* (Hs), *M*. *musculus* (Mm), *D*. *melanogaster* (Dm), *A*. *thaliana* (At), *P*. *infestans* (Pi), *C*. *lusitania* (Cl), *C*. *albicans* (Ca), *C*. *glabrata* (Cg), *S*. *cerevisiae* (Sc), *Y*. *lipolytica* (Yl), *S*. *octosporus* (So), *S*. *pombe* (Sp), *S*. *japonicus* (Sj), *P*. *graminis* (Pg), *U*. *maydis* (Um) and *C*. *neoformans* (Cn) were retrieved from UniProtKB and aligned using Clustal Omega. The tree was constructed from the alignment data obtained through Neighbourhood joining method using Simple Phyogeny and iTOL (Interactive Tree Of Life). The position of Aurora B kinase from *C*. *neoformans* is marked in red. **B.** Schematic of the kinase domain of the Aurora kinase B homolog Ipl1 in *C*. *neoformans* and its amino acid sequence conservation across species. The conserved residues are shaded, and the conservation score is color-coded in which black, green and grey correspond to the highly, moderately and poorly conserved residues respectively.
(TIF)

S2 Fig Dynamics of Ipl1 localization and nuclear envelope breakdown during cell cycle. **A.** CNNV112 cells co-expressing mCherry-Ipl1 and GFP-PCNA depicting localization of Ipl1 and PCNA respectively in the cytoplasm and in the nucleus during mitosis. Bar, 5μm (Right). **B.** CNNV112 cells co-expressing mCherry-Ipl1 and GFP-PCNA depicting localization of PCNA in the cytoplasm in the presence and absence of Ipl1 during mitosis. Bar, 5μm.
(TIF)

S3 Fig Spatio-temporal regulation of kinetochore-microtubule interactions is maintained by Ipl1. **A.** Images of CNNV114 cells co-expressing *GAL7*p-mCherry-Ipl1 and histone H4 GFP grown under non-permissive conditions for the indicated time. Bar, 5μm. **B.** The level of tubulin expression in cell lysates prepared from *GAL7*p-*IPL1* expressing cells before (0 h) and after the indicated time of incubation in non-permissive media conditions. Western blot analysis was done using anti-GFP and anti-PSTAIRE antibodies. **C.** Quantification of budding index in wild-type and Ipl1-depleted cells having unclustered kinetochores (n = 30). Mean and SEM are marked; p<0.0001, unpaired *t*-test. **D.** Distribution of cells displaying variability in kinetochore clustering timing, duration of nuclear migration and MT integrity in Ipl1-depleted conditions. Each row represents a cell indicating the color-coded status of these quantities. **E.** Localization of outer kinetochore proteins in the wild-type and Ipl1-depleted cells expressing mCherry-Mtw1 and GFP-Dad1. Bar, 5μm.
(TIF)

S4 Fig An effective cortical bias generated due to the cortical recruitment of dynein and cytoplasmic microtubules are essential for kinetochore clustering. **A.** Images of CNVY113 budded cells expressing CENP-A-GFP grown in the absence or presence of nocodazole (1μg/ml) for 1 h. Bar, 10μm. **B.** Quantification of the budding indices of CNVY113 budded cells having unclustered kinetochores upon treatment with nocodazole (n>22).; p<0.0001, unpaired *t*-test. **C.** Images of unclustered kinetochores (GFP-CENP-A) in the wild-type and cells overexpressing Dyn1. The kinetochores remained unclustered in the mutant at a higher budding index than the wild-type. Bar, 5μm. **D.** Quantification of the budding indices of budded cells of CNNV111 having unclustered kinetochores upon their growth in permissive conditions.; p<0.05, unpaired *t*-test. **E.** Images of unclustered kinetochores (GFP-CENP-A) in the wild-type and cells with reduced Dyn1. The kinetochores remained unclustered in the mutant at a higher budding index than the wild-type. Bar, 5μm. **F.** Quantification of the budding indices of budded cells of CNNV111 having unclustered kinetochores upon their growth in non-permissive conditions.; p<0.05, unpaired *t*-test.
(TIF)

S5 Fig Heterogeneously perturbed microtubules partially recapitulate defective MTOC clustering and improper nuclear migration in Ipl1-depleted cells. **A-B.** Simulated results correspond to homogeneously perturbed cMTs. **A.** Simulated characteristics of MTOC clustering time corresponding to an average cMT length. The horizontal solid green line depicts the mean MTOC clustering time (n>25), and vertical solid green line represents the mean cMT length (n>250) obtained from the experiment. For solid lines, perpendicular dotted lines estimate other axis parameters for each curve. **B.** The nuclear migration time corresponding to the characteristic average cMT length. As described in A, solid green lines correspond to experimental data (n>30) while perpendicular dotted lines estimate the projected outcome relevant to the remaining axis. **C.** Distribution of the neck to nucleus distance in the presence of heterogeneously perturbed cMTs experiencing uniform bias at the cortex. Mean of the distributions shown in the inset. -ve distance indicates that nucleus is inside the mother. **D.** Distribution of the neck to nucleus distance in the presence of heterogeneous cMTs coupled with the length dependent bias (n = 18). Mean of the distributions shown in the inset. -ve distance indicates that nucleus is inside the mother.
(TIF)

S6 Fig Localization of dynein upon depletion of Ipl1. Representative images depicting localization of Dyn1 in the CNNV116 cells at the unbudded and small-budded stages expressing Dyn1-3xGFP upon their growth in the permissive and non-permissive conditions. All the images in this panel were processed at the same microscopy settings and were analyzed after deconvolution. Bar, 10μm.
(TIF)

S1 Table Strains used in this study. (DOCX)

S2 Table Primers used in this study. (DOCX)

S1 Movie Timing of localization of Ipl1 in the cytoplasm and nucleus with nuclear migration. The nucleus is visualized by GFP-tagged PCNA. GFP-PCNA leaks out into the cytoplasm upon nuclear envelope breakdown. Ipl1 is localized with mCherry. Images were acquired every 120s with confocal microscopy.
(AVI)

S2 Movie Nuclear migration in the wild-type and Ipl1-depleted cells. The nucleus is visualized by GFP-tagged histone H4. Images were acquired every 60 s with confocal microscopy.
(AVI)

S3 Movie MTOC clustering and nuclear migration in the wild-type and *ipl1* mutant with the effect of biased cortical interaction. (A) Wild-type, and conditional *ipl1* mutant where structural stability of MTs is (B) slightly affected, (C) moderately affected and (D) highly affected.
(AVI)
