# Supplementary figures and images for: Spatio-temporal regulation of nuclear division by Aurora B kinase Ipl1 in Cryptococcus neoformans
Source: PLoS Genet. 2019 Feb 14;15(2):e1007959. doi: 10.1371/journal.pgen.1007959 (PMC6392335; doi:10.1371/journal.pgen.1007959)

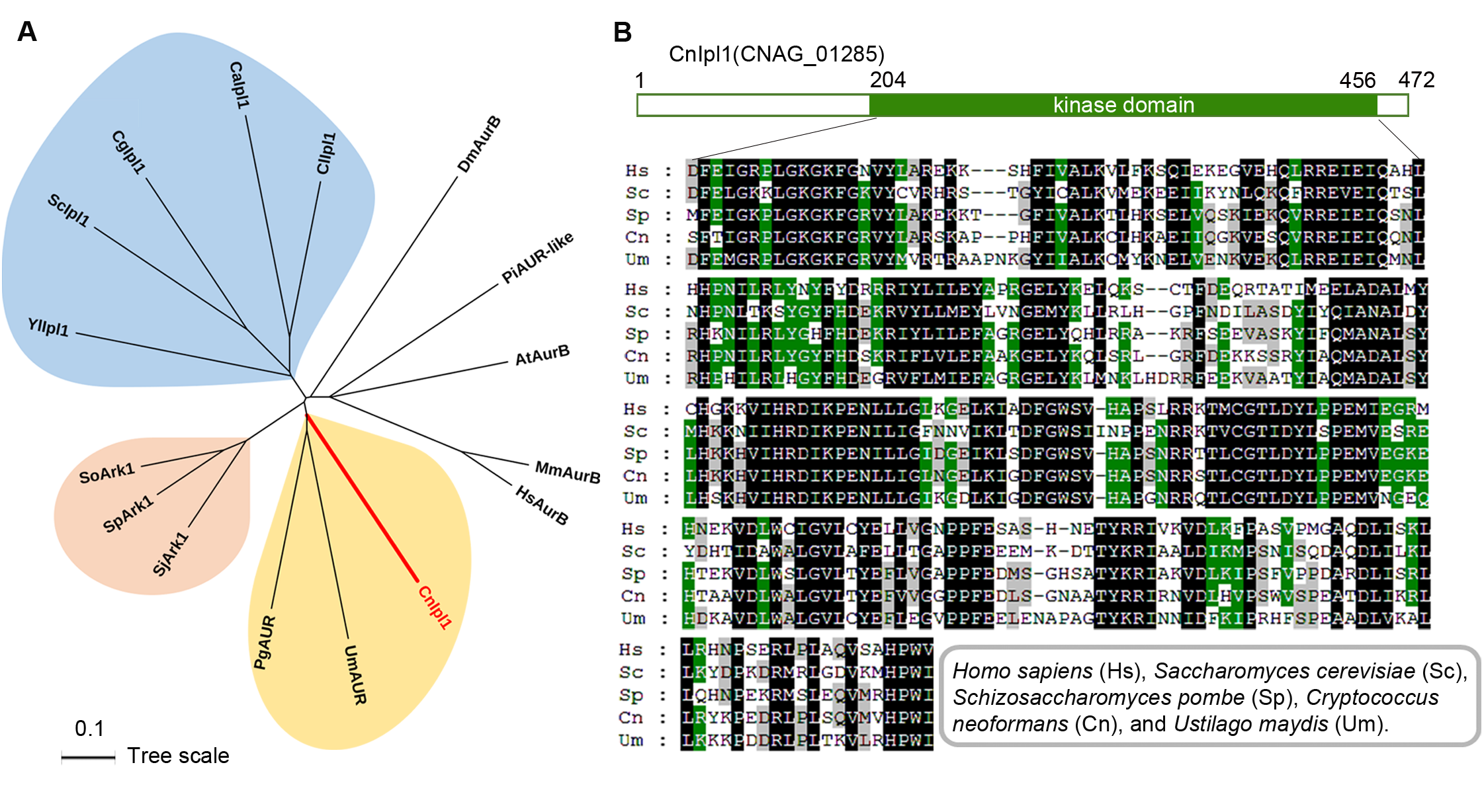

Supplement: S1 Fig — A. Phylogenetic analysis of Aurora kinases. Amino acid sequences for Aurora kinases in H. sapiens (Hs), M. musculus (Mm), D. melanogaster (Dm), A. thaliana (At), P. infestans (Pi), C. lusitania (Cl), C. albicans (Ca), C. glabrata (Cg), S. cerevisiae (Sc), Y. lipolytica (Yl), S. octosporus (So), S. pombe (Sp), S. japonicus (Sj), P. graminis (Pg), U. maydis (Um) and C. neoformans (Cn) were retrieved from UniProtKB and aligned using Clustal Omega. The tree was constructed from the alignment data obtained through Neighbourhood joining method using Simple Phyogeny and iTOL (Interactive Tree Of Life). The position of Aurora B kinase from C. neoformans is marked in red. B. Schematic of the kinase domain of the Aurora kinase B homolog Ipl1 in C. neoformans and its amino acid sequence conservation across species. The conserved residues are shaded, and the conservation score is color-coded in which black, green and grey correspond to the highly, moderately and poorly conserved residues respectively. (TIF) [file pgen.1007959.s001.tif]

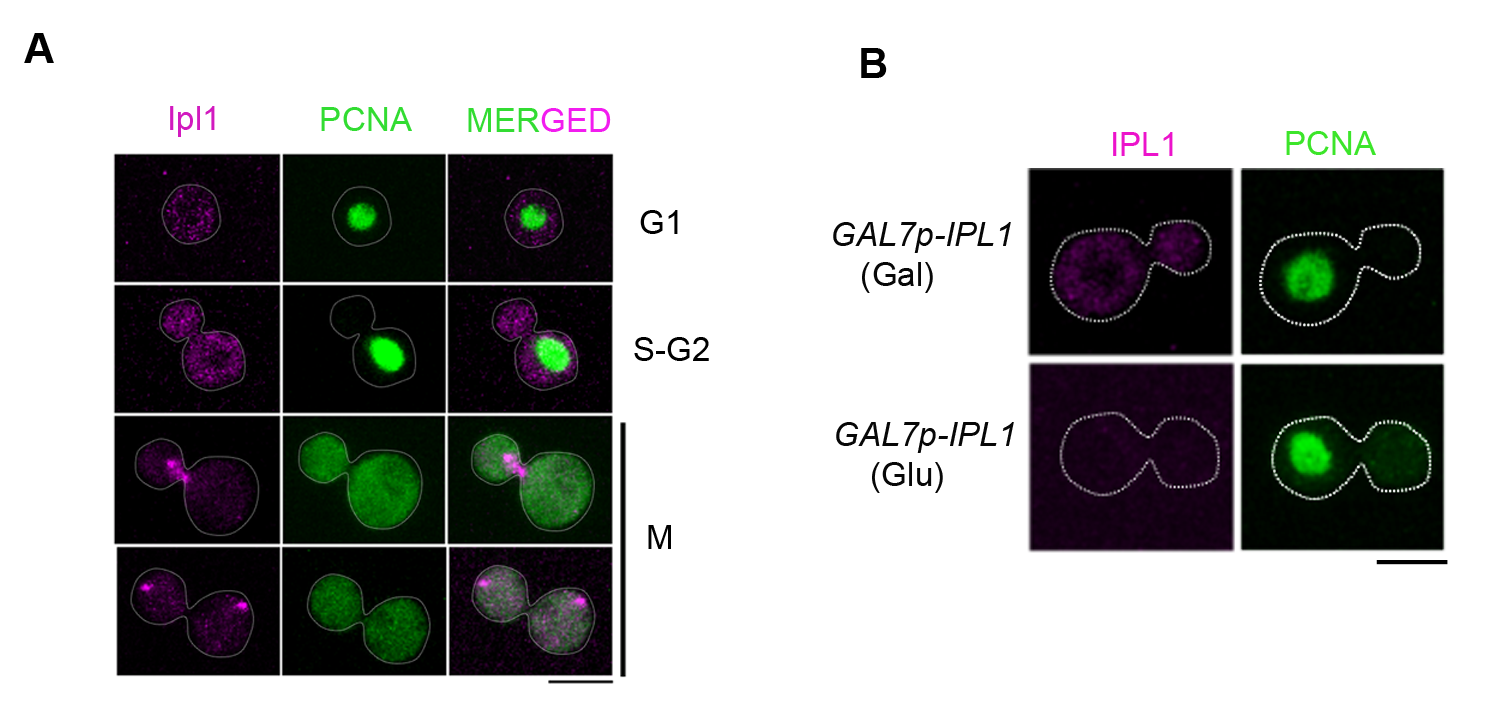

Supplement: S2 Fig — A. CNNV112 cells co-expressing mCherry-Ipl1 and GFP-PCNA depicting localization of Ipl1 and PCNA respectively in the cytoplasm and in the nucleus during mitosis. Bar, 5μm (Right). B. CNNV112 cells co-expressing mCherry-Ipl1 and GFP-PCNA depicting localization of PCNA in the cytoplasm in the presence and absence of Ipl1 during mitosis. Bar, 5μm. (TIF) [file pgen.1007959.s002.tif]

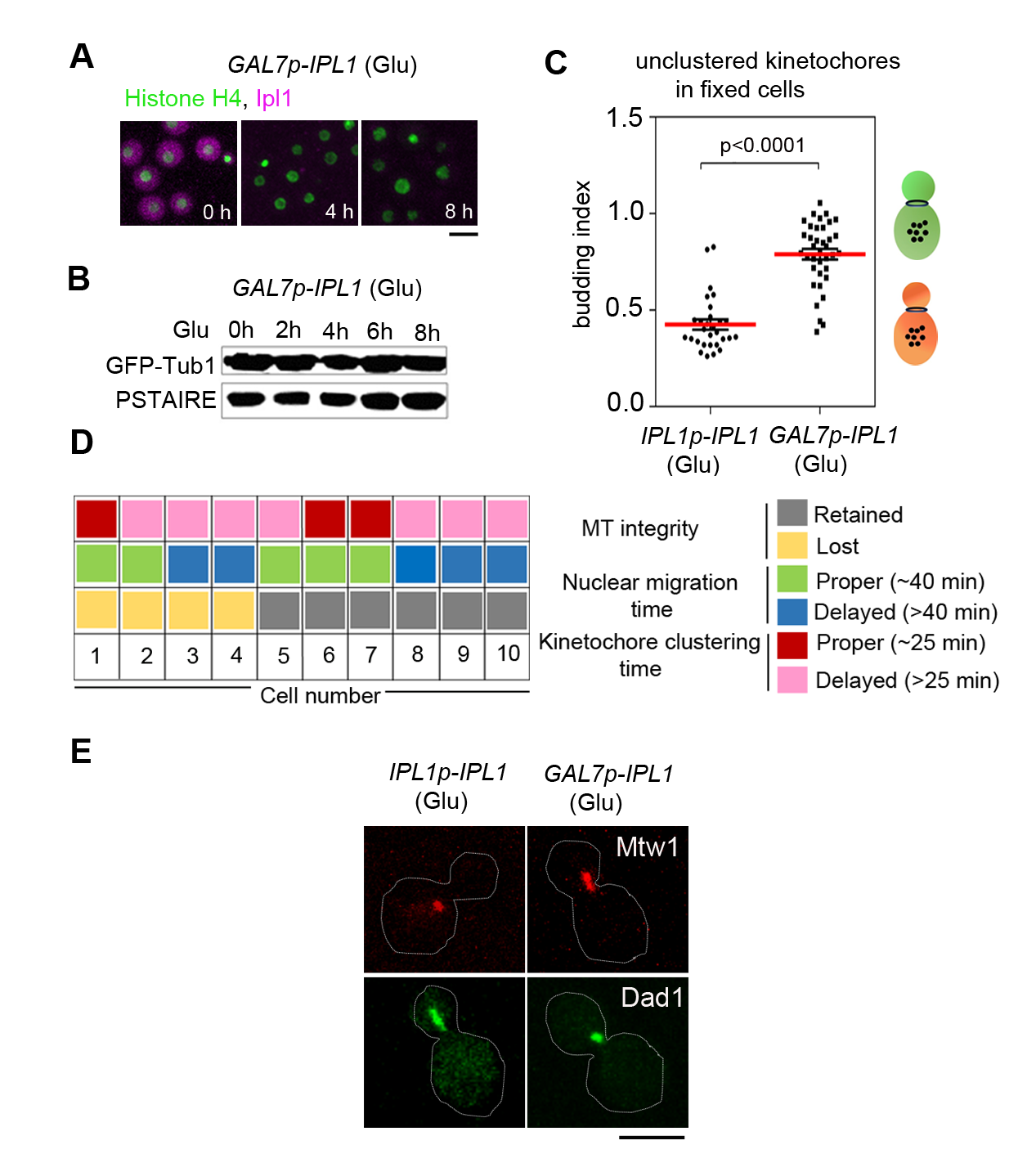

Supplement: S3 Fig — A. Images of CNNV114 cells co-expressing GAL7p-mCherry-Ipl1 and histone H4 GFP grown under non-permissive conditions for the indicated time. Bar, 5μm. B. The level of tubulin expression in cell lysates prepared from GAL7p-IPL1 expressing cells before (0 h) and after the indicated time of incubation in non-permissive media conditions. Western blot analysis was done using anti-GFP and anti-PSTAIRE antibodies. C. Quantification of budding index in wild-type and Ipl1-depleted cells having unclustered kinetochores (n = 30). Mean and SEM are marked; p<0.0001, unpaired t-test. D. Distribution of cells displaying variability in kinetochore clustering timing, duration of nuclear migration and MT integrity in Ipl1-depleted conditions. Each row represents a cell indicating the color-coded status of these quantities. E. Localization of outer kinetochore proteins in the wild-type and Ipl1-depleted cells expressing mCherry-Mtw1 and GFP-Dad1. Bar, 5μm. (TIF) [file pgen.1007959.s003.tif]

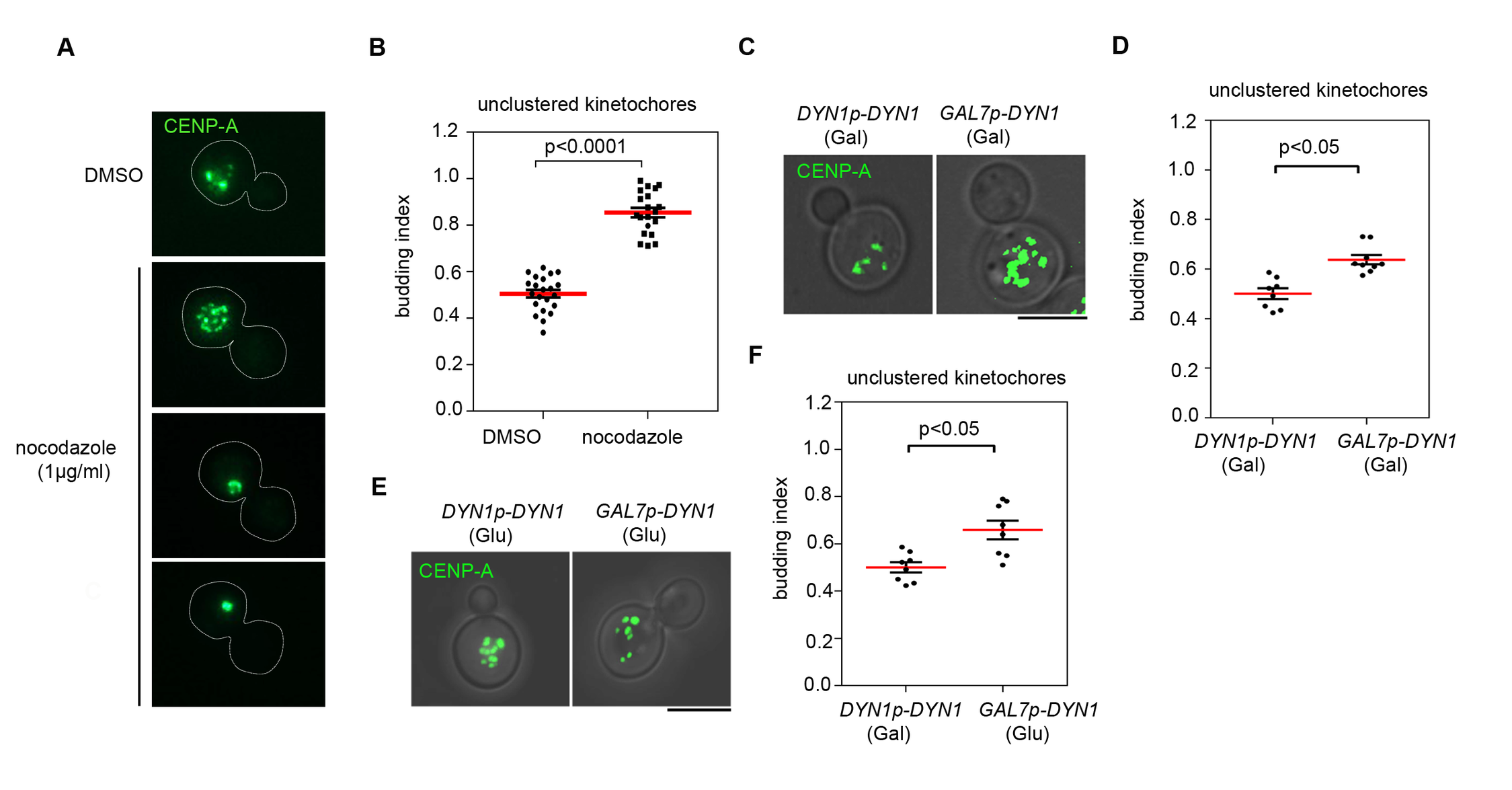

Supplement: S4 Fig — A. Images of CNVY113 budded cells expressing CENP-A-GFP grown in the absence or presence of nocodazole (1μg/ml) for 1 h. Bar, 10μm. B. Quantification of the budding indices of CNVY113 budded cells having unclustered kinetochores upon treatment with nocodazole (n>22).; p<0.0001, unpaired t-test. C. Images of unclustered kinetochores (GFP-CENP-A) in the wild-type and cells overexpressing Dyn1. The kinetochores remained unclustered in the mutant at a higher budding index than the wild-type. Bar, 5μm. D. Quantification of the budding indices of budded cells of CNNV111 having unclustered kinetochores upon their growth in permissive conditions.; p<0.05, unpaired t-test. E. Images of unclustered kinetochores (GFP-CENP-A) in the wild-type and cells with reduced Dyn1. The kinetochores remained unclustered in the mutant at a higher budding index than the wild-type. Bar, 5μm. F. Quantification of the budding indices of budded cells of CNNV111 having unclustered kinetochores upon their growth in non-permissive conditions.; p<0.05, unpaired t-test. (TIF) [file pgen.1007959.s004.tif]

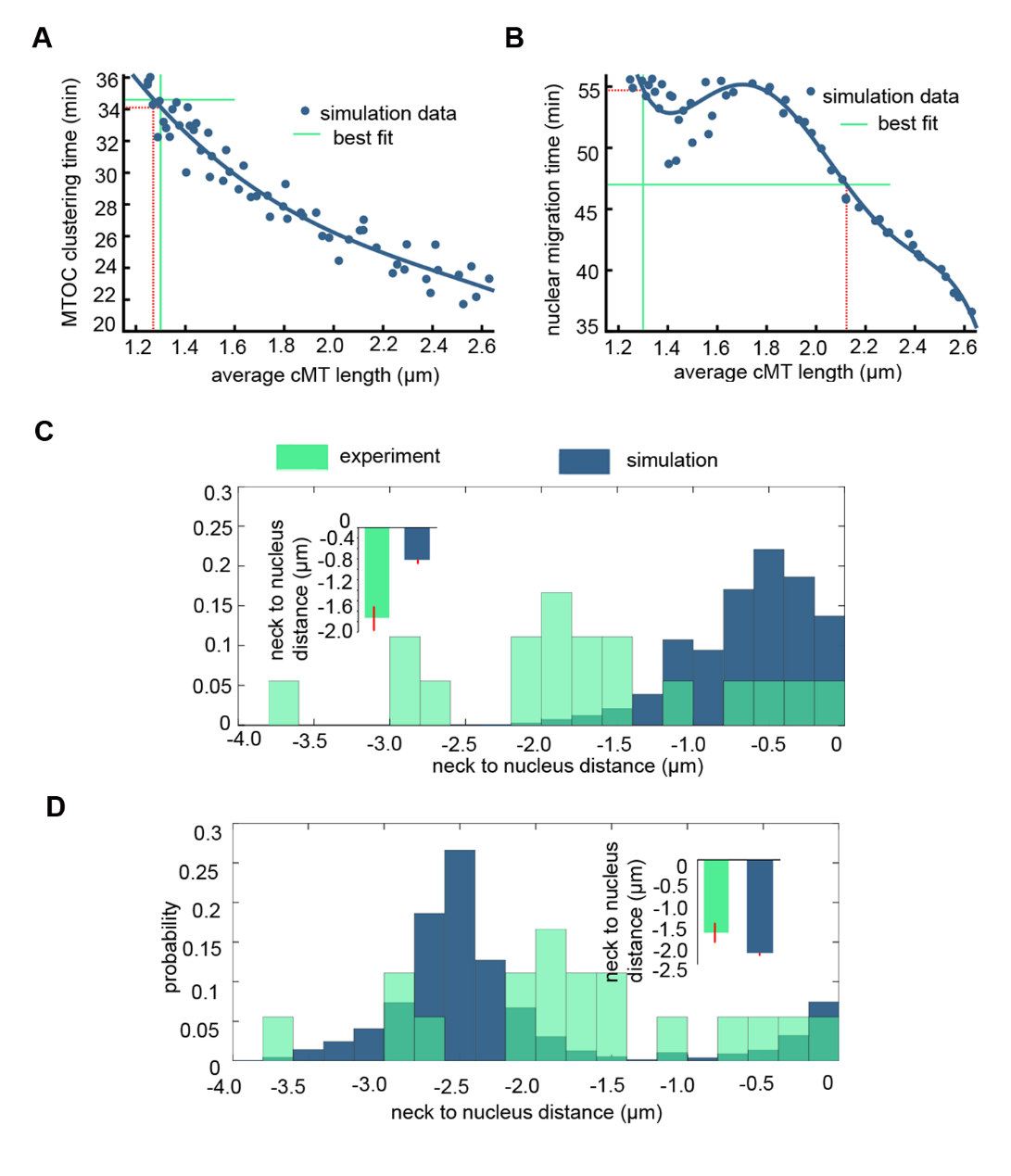

Supplement: S5 Fig — A-B. Simulated results correspond to homogeneously perturbed cMTs. A. Simulated characteristics of MTOC clustering time corresponding to an average cMT length. The horizontal solid green line depicts the mean MTOC clustering time (n>25), and vertical solid green line represents the mean cMT length (n>250) obtained from the experiment. For solid lines, perpendicular dotted lines estimate other axis parameters for each curve. B. The nuclear migration time corresponding to the characteristic average cMT length. As described in A, solid green lines correspond to experimental data (n>30) while perpendicular dotted lines estimate the projected outcome relevant to the remaining axis. C. Distribution of the neck to nucleus distance in the presence of heterogeneously perturbed cMTs experiencing uniform bias at the cortex. Mean of the distributions shown in the inset. -ve distance indicates that nucleus is inside the mother. D. Distribution of the neck to nucleus distance in the presence of heterogeneous cMTs coupled with the length dependent bias (n = 18). Mean of the distributions shown in the inset. -ve distance indicates that nucleus is inside the mother. (TIF) [file pgen.1007959.s005.tif]

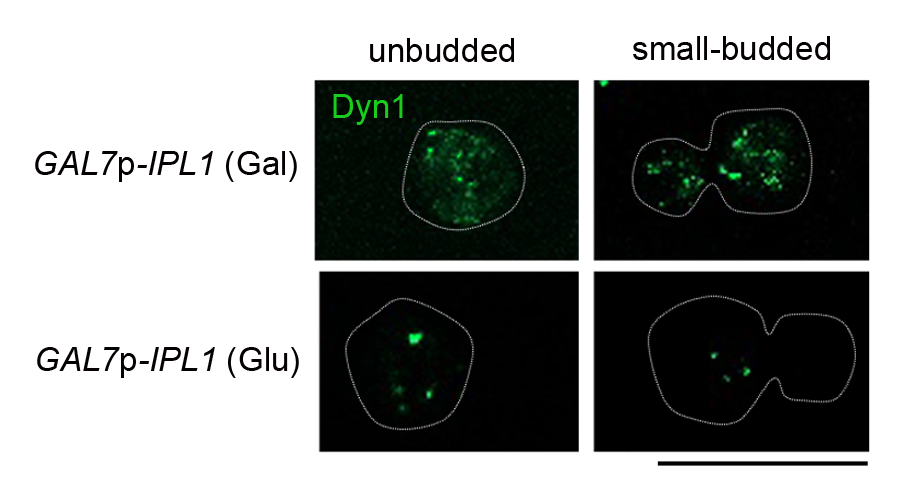

Supplement: S6 Fig — Representative images depicting localization of Dyn1 in the CNNV116 cells at the unbudded and small-budded stages expressing Dyn1-3xGFP upon their growth in the permissive and non-permissive conditions. All the images in this panel were processed at the same microscopy settings and were analyzed after deconvolution. Bar, 10μm. (TIF) [file pgen.1007959.s006.tif]
