# Supplementary material for: Spatio-temporal regulation of nuclear division by Aurora B kinase Ipl1 in Cryptococcus neoformans
Source: PLoS Genet. 2019 Feb 14;15(2):e1007959. doi: 10.1371/journal.pgen.1007959 (PMC6392335; doi:10.1371/journal.pgen.1007959)
Supplement: S1 Table — (DOCX) [file pgen.1007959.s007.docx]

**S1 Table. Strains used in this study**

| Strain name | Genotype | Reference |
| --- | --- | --- |

| H99 | Wild-type | [66] |
| --- | --- | --- |
| CNNV101 | MATα IPL1::GAL7p-IPL1-HygB | This study |
| CNNV102 | MATα IPL1:: GAL7p-IPL1-HygB | This study |
| CNNV103 | MATα IPL1:: GAL7p-mCherry-IPL1-HygB | This study |
| CNVY108 | MATα H99::GFP-H4-NAT (pVY3) | [20] |
| CNNV104 | MATα H99::GFP-H4-NAT (pVY3), IPL1::GAL7p-IPL1-HygB | This study |
| CNVY107 | MATa H99::GFP-αTUB1-NAT (pLKB35), H99::mCherry-CSE4-NEO (pLKB74) | This study |
| CNNV105 | MATa H99::GFP-αTUB1-NAT (pLKB35), H99::mCherry-CENP-A-NEO (pLKB74), IPL1:: GAL7p-IPL1-HygB | This study |
| CNNV107 | MATα H99::GFP-H4-NAT (pLKB35) + *BIM1Δ::NEO* | This study |
| CNVY103 | MATa MTW1::MTW1-mCherry-NEO (pLK25) | [20] |
| CNNV108 | MATa MTW1::MTW1-mCherry-NEO (pLK25), GAL7p-IPL1:HygB | This study |
| CNVY120 | MATa H99::GFP-DAD1-NAT (pVY2), H99:: mCherry-CENP-A-NEO (pLKB74) | [20] |
| CNNV109 | MATa H99::GFP-DAD1:NAT (pVY2), H99:: mCherry-CENP-A-NEO (pLKB74), IPL1::GAL7p-IPL1-HygB | This study |
| CNVY113 | MATα H99::GFP-CENP-A-NAT (pVY1) | This study |
| CNNV110 | MATα H99::GFP-H4-NAT (pVY3)*,* *DYN1*:: GAL7p-DYN1-HygB | This study |
| CNNV111 | MATα H99::GFP-CENP-A-NAT (pVY1)*,* DYN1::GAL7p-DYN1-HygB | This study |
| CNNV112 | MATα H99::GFP-PCNA-NAT (pCIN19), IPL1::GAL7p-mCherry-IPL1-HygB | This study |
| CNNV113 | MATα IPL1:: IPL1p-IPL1-3xGFP-NEO | This study |
| CNNV114 | MATα H99::GFP-H4-NAT (pVY3), IPL1::GAL7p-mCherry-IPL1-HygB | This study |
| CNNV116 | MATα DYN1:: DYN1p-DYN1-3xGFP-NEO, IPL1::GAL7p-IPL1-HygB | This study |
| CNNV118 | MATα SPC98::SPC98p-SPC98-3xGFP-NEO, IPL1::GAL7p-IPL1-HygB | This study |
| CNNV119 | MATα BIM1::BIM1p-BIM1-3xGFP-NEO | This study |
