# Supplementary material for: Spatio-temporal regulation of nuclear division by Aurora B kinase Ipl1 in Cryptococcus neoformans
Source: PLoS Genet. 2019 Feb 14;15(2):e1007959. doi: 10.1371/journal.pgen.1007959 (PMC6392335; doi:10.1371/journal.pgen.1007959)
Supplement: S2 Table — (DOCX) [file pgen.1007959.s008.docx]

**S2 Table. Primers used in this study**

| Primer name | Sequence | Description |
| --- | --- | --- |
| NV117 | CAGCTCGAGATG TCC TCG CAG AAT GTT TC | Forward cloning primer for downstream of *GAL7*p-*IPL1* cassette |
| NV118 | AGTGGTACCTTG CCT TCC ACC ATC TCA GG | Reverse cloning primer for downstream of *GAL7*p-*IPL1* cassette |
| NV116 | ATTAGCGGCCGCGGA GGC CAA ATC TGG AGG | Forward cloning primer for upstream of *GAL7*p-*IPL1* cassette |
| NV123 | ATTTGCGGCCGCTAT GAC CTA GAA CTG ACA GAC T | Reverse cloning primer for upstream of *GAL7*p-*IPL1* cassette |
| Gal7pr F | ATACGCGAGTCCAACTTCGC | Forward primer for confirmation of integration of *GAL7*p-*IPL1* cassette |
| NV128 | CCG GTA GGT AGC GGC GTT TCC | Reverse primer for confirmation of integration of *GAL7*p-*IPL1* cassette |
| NV349 | CACCGGTCATAACCTATCGCTCC | Forward primer for the first overlap PCR fragment of *BIM1* deletion cassette |
| NV350 | CGTGTTAATACAGATAAACCCAAGGAGACGTATCGCAGTG | Reverse primer for the first overlap PCR fragment of *BIM1* deletion cassette |
| NV351 | CACTGCGATACGTCTCCTTGGGTTTATCTGTATTAACACG | Forward primer for the second overlap PCR fragment of *BIM1* deletion cassette |
| NV352 | GACAAAGGGAGAAACGATGGCTGCGAGGATGTGAGC | Reverse primer for the second overlap PCR fragment of *BIM1* deletion cassette |
| NV353 | GCTCACATCCTCGCAGCCATCGTTTCTCCCTTTGTC | Forward primer for the third overlap PCR fragment of *BIM1* deletion cassette |
| NV354 | ATCAATGTCGGCACACATCACC | Reverse primer for the third overlap PCR fragment of  *BIM1* deletion cassette |
| NV370 | TAAGGTGGATTAGTGGAGGA | Forward primer for confirmation of *BIM1* deletion |
| NV371 | GAAGAGGGTGGGTCTGAAGG | Reverse primer for confirmation of *BIM1* deletion |
| NV404 | ATTTGCGGCCGCTGAGATCATGGTCGCTTACATC | Forward cloning primer for downstream of *GAL7*p-*DYN1* cassette |
| NV358 | ATTTGCGGCCGCATAAAAGCAAAAAGGCAGCGAC | Reverse cloning primer for downstream of *GAL7*p-*DYN1* cassette |
| NV355 | CGCGGATCCATGACAATGGCCTCGTC | Forward cloning primer for upstream of *GAL7*p-*DYN1* cassette |
| NV356 | ACGCGTCGACAGTCCTTCATCAAGATGTTATGC | Reverse cloning primer for upstream of *GAL7*p-*DYN1* cassette |
| NV405 | CTAGACACTCTTATCAGCACCATCC | Reverse primer for confirmation of integration of *GAL7*p-*DYN1* |
| NV132 | CGCGGATCCATGGTGAGCAAGGGC | Forward primer for N-terminal tagging of Ipl1 with mCherry |
| NV133 | CAGCTCGAGCTTGTACAGCTCGTCCAT | Reverse primer for N-terminal tagging of Ipl1 with mCherry |
| SHR490 | CTGAGGATCCGGTGCCGGCGCTCTGGAAGCACGTGTAAAAC | Forward primer for N-terminal tagging of PCNA with GFP |
| SHR491 | CTGAGGATCCACTAGTTGAGAACTCCGAGTTCGTG | Reverse primer for N-terminal tagging of PCNA with GFP |
| NV430 | CAGCTCGAGCCATTATCCCTACCGTCG | Forward cloning primer for downstream of *Ipl1*p-*IPL1*-3X cassette |
| NV431 | CCCATCGATGCTGACTTCCTGATACCGCTA | Reverse cloning primer for downstream of *Ipl1*p-*IPL1*-3X cassette |
| NV436 | TCCCCGCGGTTTTTCAAGCCTATGCACCTA | Forward cloning primer for upstream of *Ipl1*p-*IPL1*-3X cassette |
| NV437 | TCCCCGCGGTAACAATAGTAGTGTCAATCACATACA | Reverse cloning primer for upstream of *Ipl1*p-*IPL1*-3X cassette |
| NV432 | ATTAGCGGCCGCGGTTTATCTGTATTAACACGGAAGA | Forward cloning primer for Neomycin *(NEO)* of *Ipl1*p-*IPL1*-3X cassette |
| NV433 | ATTAGCGGCCGCGCTGCGAGGATGTGAGC | Reverse cloning primer for Neomycin *(NEO)* of *Ipl1*p-*IPL1*-3X cassette |
| NV452 | AGTGGTACCAACGTCGATCCTGTTCAGAT | Forward cloning primer for upstream of *Dyn1*p-*DYN1*-3X cassette |
| NV453 | CCCATCGATGCCGCGGCAGTCAAACATACA | Reverse cloning primer for upstream of *Dyn1*p-*DYN1*-3X cassette |
| NV454 | ATCGAGCTCTAAATCTAGAAGAGGGACAGG | Forward cloning primer for downstream of *Dyn1*p-*DYN1*-3X cassette |
| NV455 | ATCGAGCTCACTTTATGCTCATTGGCG | Reverse cloning primer for downstream of *Dyn1*p-*DYN1*-3X cassette |
| NV478 | AGTGGTACCTCTGGAGGTTGAAGCGTG | Forward cloning primer for upstream of *Spc98*p-*SPC98*-3X cassette |
| NV479 | CCCATCGATGCAGCCCTCGCCGACC | Reverse cloning primer for upstream of *Spc98*p-*SPC98*-3X cassette |
| NV477 | ATCGAGCTCCTACCGGTCTCACACC | Forward cloning primer for downstream of *Spc98*p-*SPC98*-3X cassette |
| NV476 | ATCGAGCTCTAGGATATAGGTTTTCGGGATAATAA | Reverse cloning primer for downstream of *Spc98*p-*SPC98*-3X cassette |
| NV470 | AGTGGTACCCCAGCGTACGTCGGAG | Forward cloning primer for upstream of *Bim1*p-*BIM1*-3X cassette |
| NV471 | CCCATCGATGCAAAAGTCTCTTCCTCACCCC | Reverse cloning primer for upstream of *Bim1*p-*BIM1*-3X cassette |
| NV472 | ATCGAGCTCCGTTTCTCCCTTTGTCCC | Forward cloning primer for downstream of *Bim1*p-*BIM1*-3X cassette |
| NV473 | ATCGAGCTCTCTAGAAAAGCAGGTCGTCAGTCTT | Reverse cloning primer for downstream of *Bim1*p-*BIM1*-3X cassette |
